# Sustained and Enhanced Nucleate Boiling Using Hierarchical Architectures at Large Superheats

**DOI:** 10.1002/EXP.20240137

**Published:** 2025-08-25

**Authors:** Ji‐Xiang Wang, Hongmei Wang, Christopher Salmean, Binbin Cui, Ming‐Liang Zhong, Yufeng Mao, Jia‐Xin Li, Shuhuai Yao

**Affiliations:** ^1^ Department of Mechanical and Aerospace Engineering The Hong Kong University of Science and Technology Hong Kong SAR P. R. China; ^2^ Hebei Key Laboratory of Man‐machine Environmental Thermal Control Technology and Equipment Hebei Vocational University of Technology and Engineering Xingtai P. R. China; ^3^ Shanghai Golden Deep Ltd. Shanghai P. R. China; ^4^ Department of Electrical and Computer Engineering University of Toronto Toronto Ontario Canada; ^5^ Institute of Optics and Electronics Chinese Academy of Sciences Chengdu P. R. China; ^6^ National Key Laboratory of Optical Field Manipulation Science and Technology Chinese Academy of Sciences Chengdu P. R. China; ^7^ Beijing Institute of Astronautics System Engineering Beijing P. R. China

**Keywords:** deep learning, droplet boiling, Leidenfrost delay, multiphase fluids, nano–micro hierarchical structure

## Abstract

Droplet boiling is a common occurrence in many industrial processes, but it can be hindered by the Leidenfrost effect. The Leidenfrost point (LP), defined as the temperature at which an accumulated and stagnant vapor forms between the liquid and the heated solid, consequently deteriorates cooling performance. In this study, inspired by nature, we demonstrate how using a nano‐micro hierarchical triple‐passage architecture with a higher aspect ratio enhances both vapor and liquid spreading dynamics, boosts heat transfer, and thus elevates the LP. Our results show that the LP is promoted to 273°C, which is a delay of approximately 130°C compared to the LP of 145°C on a copper surface. Through theoretical analysis, we develop a multi‐force competition model to reveal the underlying physics of this sustained nucleate boiling. Our findings challenge traditional wisdom, indicating that lower impact velocities of a droplet, though sacrificing the convection, delay the LP through impact pattern manipulation. Additionally, we adopt a physics‐informed deep neural network framework to accurately model the nonlinear behavior of droplet boiling (from nucleate boiling to LP) on various surfaces within an ≈11% error. The results here have potential applications in designing more efficient droplet‐based boiling heat transfer devices and in controlling droplet boiling at high temperatures.

## Graphical abstract

Sustained nucleate boiling and Leidenfrost delay are critical for thermal and energy industries. Leveraging vapor buffer and reducing water‐hammer pressure, this study realizes the enhancement of nucleate boiling and postponement of Leidenfrost by rationally designing and developing hierarchical nano–micro structured surfaces. Besides, AI technology is adopted to predict droplet boiling patterns on various structured surfaces.

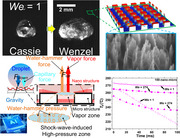

## 1 Introduction

The phase‐change boiling or evaporation of droplets on solids occurs not only in natural phenomena but also in multiple industrial processes such as extreme ultraviolet nanolithography [1], fuel droplet combustion [2], vapor and fresh water production [3, 4], materials fabrication [5], liquid manipulation [6, 7], thermal control [8, 9], aerospace vehicle thermal protection [10], heterogeneous catalysis [11], and electronics spray cooling [12]. Droplet impingement boiling occurs when subcooled or saturated droplets impact upon a hot (above the saturated temperature of the liquid) solid material [13]. A strong and complex liquid–solid–vapor interaction takes place at the contact point where part/all of the liquid‐phase can be suddenly turned into the vapor phase, and the temperature of the solid material can be abruptly decreased [14]. Understanding the kinetic and phase change behavior is therefore a persistent topic of focus in the scientific community, as it is a crucial facet for understanding and optimization of real‐world industrial processes [15, 16, 17]. Enhanced heat transfer can be obtained by such liquid–vapor boiling through both heterogeneous and homogeneous nucleate boiling and convection effects [18]. Therefore, the droplet with a high impact velocity usually possesses a maximum boiling heat transfer capability [19, 20].

However, when the solid's temperature reaches a certain threshold (≈150°C [21]), the Leidenfrost point (LP) occurs, whereby impinging droplets are insulated from the heated surface by a cushion of their own vapor, resulting in vastly deteriorated heat transfer [22]. Besides coolant development [23] and active technologies [24, 25], much effort has been made to delay the LP through enhancing solid's hydrophilicity, facilitating liquid spreading, and strengthening vapor escape [26]. Farokhnia et al. [27] and Wang et al. [28] integrated a super‐hydrophilic layer with nano‐scale structures into micro‐pillared solids to create decoupled hierarchical structures. The capillary force within the nano‐structure of the super‐hydrophilic layer promotes the liquid spreading upon the solid and micro‐pillared structures provide vapor escape channels. Although these hybrid structure shows great promise in LP delay at extremely high superheats, their low heat transfer capabilities at low superheats, due to the low thermal‐conductive super‐hydrophilic layer, prevent them from wider application [27]. Current lithography technology allows the fabrication of artificial homogenous high thermal‐conductive nano–micro hierarchical structure. Studying nature biological organism's super‐hydrophilicity is an effective approach to design and fabricate super‐hydrophilic materials. The surface of the cornea in the human eye is a classic example of a biological super‐hydrophilic surface [29]. This is because it causes tears to spread quickly across its surface, which helps to eliminate light scattering and improve visibility. Besides, fish scales have been revealed to have super‐hydrophilicity due to the liquid sucking structures [30]. Parker and Lawrence reported a desert beetle's bumpy structure, which can enhance liquid delivery [31]. Aside from structured surfaces, intrinsic super‐hydrophilicity has also been achieved through the heterogeneous distribution of hydrophilic hydroxy groups [32, 33].

Herein, by utilizing our knowledge of how multiscale structure influences super‐hydrophilicity in natural systems, we develop advanced homogenous silicon‐based micro‐bumpy material integrated with three‐dimensional (3D) super‐hydrophilic nano‐fish scale structures for super‐wetting properties. Theoretically and experimentally, this paper evaluates the use of silicon‐based nano–micro hierarchical structured surfaces (HSSs) to enhance and sustain nucleate boiling at large superheats. The HSSs' enhanced wettability, increased solid fraction, and vapor buffer effect (VBE) enable LP delay. The VBE, provided by micro‐structures with a large internal space volume, stores and buffers sudden boiling vapor, suppressing LP by regulating the vapor force. Experiments across a wide range of Weber numbers (*We*) revealed that low impact velocity further delays LP through impact pattern manipulation, differing from previous publications where larger impact velocity enhances heat transfer [19, 20, 34, 35]. A multi‐forces competition model explains the impact of *We* and VBE on LP delay upon HSSs. A physics‐informed deep neural network (DNN) model was established, learning nonlinear boiling laws across different boiling regions and surface structures. This model accurately predicts the heat transfer coefficient across broad temperature ranges and various nano–micro HSS configurations, aiding in evaluating boiling performance and predicting LP occurrence for similar nano–micro HSSs.

## 2 Results

**FIGURE 1:**
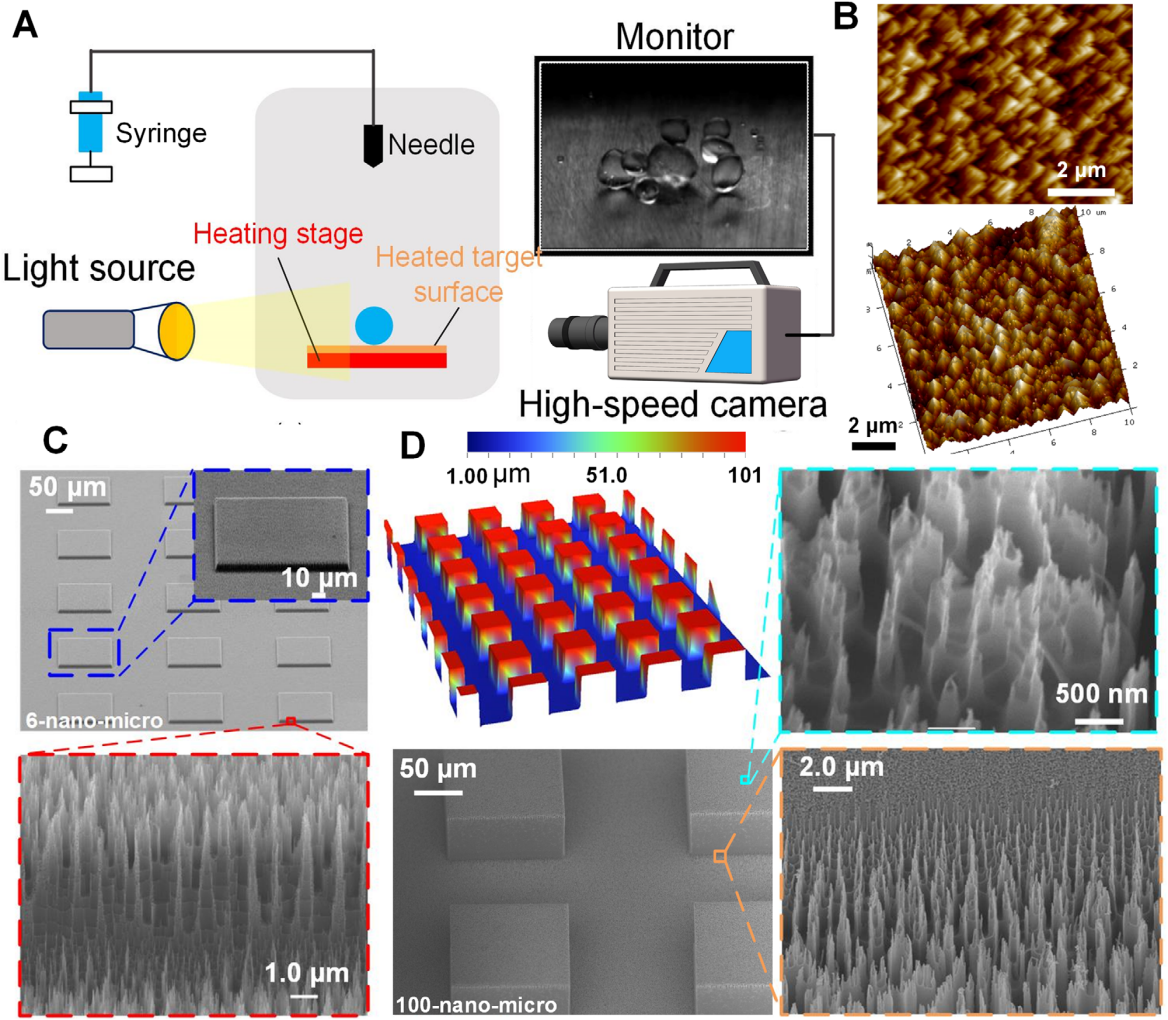
Experimental system and surface characterization. (A) Schematics of the experimental system. (B) Atomic force microscope (AFM) characterization of the nano‐fishscale. Surface architecture characterization of (C) 6‐nano‐micro and (D) 100‐nano‐micro using scanning electron microscope (SEM) and 3D measurement laser microscope techniques.

Figure 1 shows the schematics of the experimental system. A schematic diagram of the water droplet boiling experimental stage is shown in Figure 1A, and a detailed description can be found in Section S1. A heated target surface was fixed on a heating stage. There were two thermocouple measuring points on the backside of the target surface to monitor the surface temperature. As shown in Figure 1A, single droplet impact boiling tests were carried out on various utilized target surfaces with the desired surface temperature. When the droplet impacts the heated target surface, multiple heat transfer modes (conductive, convective, radiative, and phase‐change heat transfer) take place to govern the surface temperature. For the heat dissipation route, besides the heat transfer via the droplet, radiative heat transfer based on Stefan–Boltzmann law [36] should be analyzed for an overall understanding of boundary effect (refer to Figure S2 and its description). In this paper, we designed and fabricated seven types of surfaces. Surfaces with nano‐/micro‐structures were designed via biomimetic inspiration as shown in Section 2.1. The nano‐structure design is based on super‐hydrophilic fishscale [30, 37] (shown in Figure S5a) and the micro‐structure based on beetle's bumpy topography [31] (shown in Figure S5b). These seven surfaces were: (i) flat copper surface (Cu), (ii) ultra‐smooth silica surface (smooth), (iii) silicon micro‐pillared surface with 6.4 µm‐height (micro), (iv) silicon surface with the nano‐fishscale structure (nano) as shown in Figures 1B, (v) HSS with 6 µm‐height micro‐pillars and nano‐fishscale (6‐nano‐micro) as shown in Figure 1C, (vi) HSS with 100 µm‐height micro‐pillars (with higher aspect ratio) and nano‐fishscale (100‐nano‐micro) as shown in Figure 1D, and (vii) HSS with 50 µm‐height micro‐pillars and nano‐fishscale (50‐nano‐micro). The 6‐nano‐micro, displayed in Figure 1C, was subject to the bumpy topography design. The short, bumpy micro‐pillars in the 6‐nano‐micro design allow the nano‐fishscale on both the pillar edge and intra‐pillar areas to be imaged in a single view, as marked in red in Figure 1C. The taller, bumpy micro‐pillars in the 100‐nano‐micro design necessitate the use of multiple images to show the nano‐fishscale on top of the micro‐pillars and in the intra‐pillar region, as shown in Figure 1D. The 50‐nano‐micro was utilized for model validation in this paper. Not only biomimetic design principles, these HSSs were also subject to engineering purposes for enhanced heat transfer (see Section S3.2). Procedures for fabricating these surfaces are described in Section S4.1. The SEM image of the Cu surface is displayed in Section S4.2.

**FIGURE 2:**
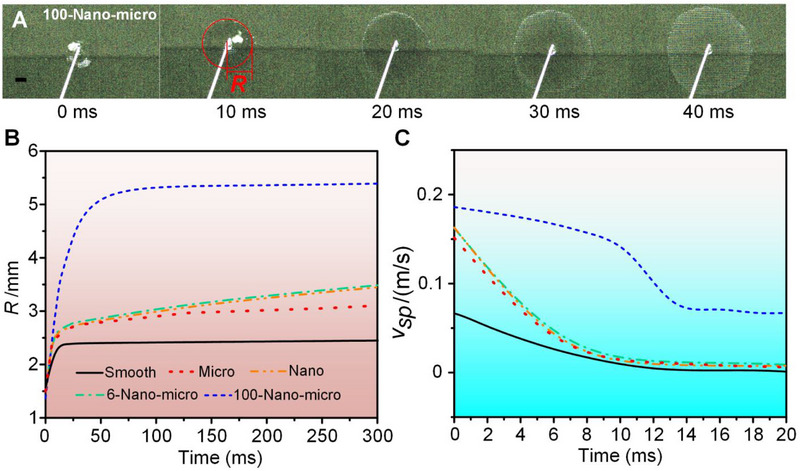
Wettability characterization of the utilized surfaces (Scale bar: 1 mm). (A) Wetting dynamic images of the 100‐nano‐micro surfaces. (B) The spreading radii, *R* (demonstrated in A), upon the utilized surfaces (smooth, micro, nano, 6‐nano‐micro, and 100‐nano‐micro). (C) Transient spreading velocities, $v_{sp}=R^{\prime }$, of the same surfaces.

The contact angles of the Cu (82.9 ± 1°), smooth (37.3 ± 1°), and micro (11.6 ± 1°) surfaces are displayed in Figure S4a–c, indicating that these surfaces can be classified as hydrophilic. The other three surfaces (nano, 6‐nano‐micro, and 100‐nano‐micro) are super‐hydrophilic [38], as characterized by spreading dynamics at room temperature (20°C), as shown in Figure S4d and Figure 2. Figures S4d and S2a show the spreading dynamics on the nano and 100‐nano‐micro surfaces; the HSS improves the spreading capability significantly, with the spreading radius of the 100‐nano‐micro surface at 40 ms exceeding that of the nano surface even after 375 ms. Figure 2B,C illustrates the spreading radii (*R*) and velocities ($R^{\prime }=v_{\mathrm{sp}}$) of all the utilized silicon‐based hydrophilic surfaces. The wetting capabilities of the nano and 6‐nano‐micro surfaces are similar due to the low aspect ratio of the micro‐pillars on the 6‐nano‐micro design, a behavior which was engendered by design in order to decouple the micro‐structure effect from the wettability property. The 100‐nano‐micro surface has superior wetting capability, with the fastest‐spreading liquid as shown in Figure 2C. However, as shown in Figure 2B, the liquid on this surface has very little subsequent horizontal spreading after 100 ms. It is thought that the liquid on this surface tends to spread vertically owing to its relatively high aspect ratio, and horizontal motion is therefore secondary. Please refer to Section S9 for the wettability characterization of the 50‐nano‐micro.

**FIGURE 3:**
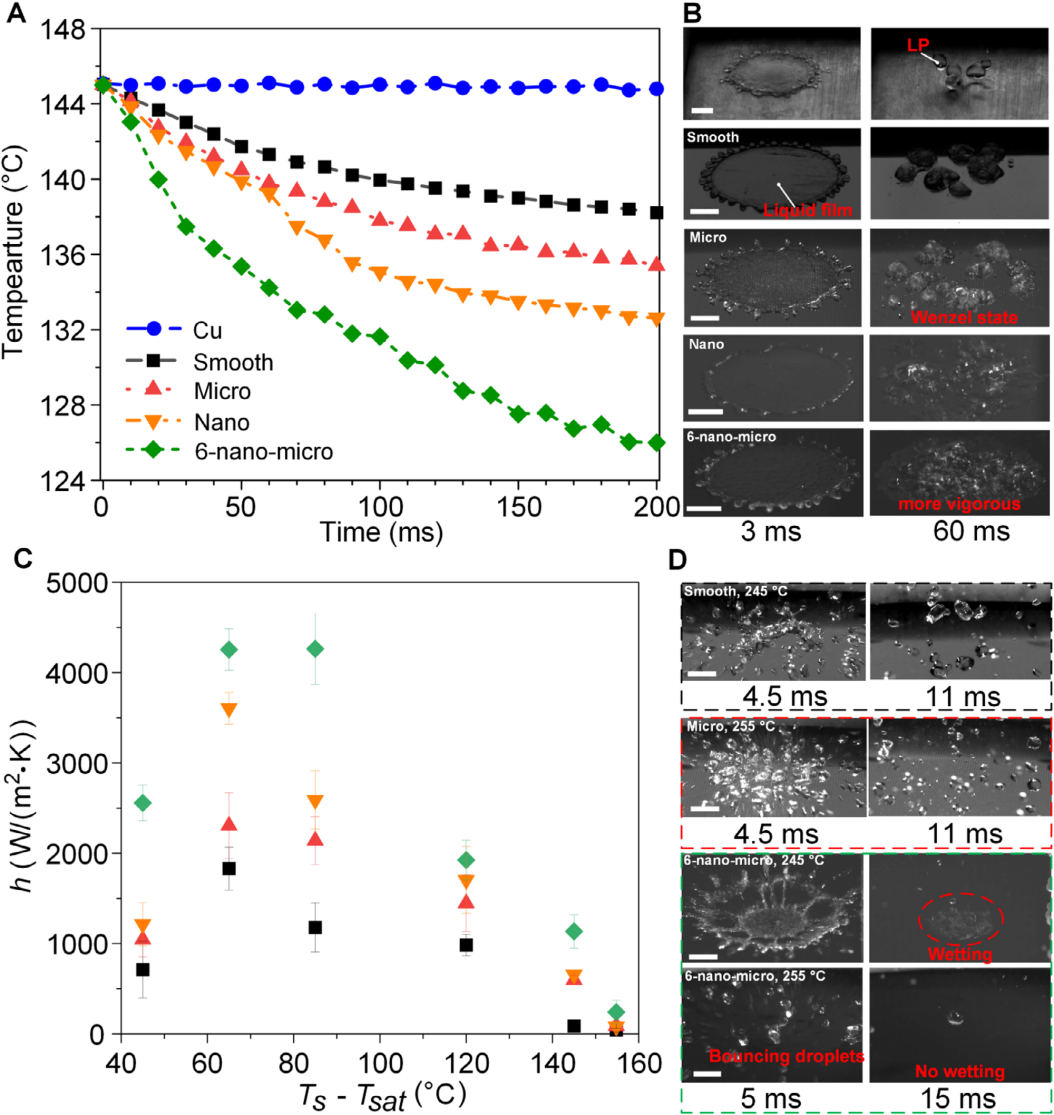
Droplet (*We* = 274) boiling behavior upon multiple materials at different surface temperatures. (A) Temperature trends during impact at surface temperature *T*_s_ of 145°C (surface superheat: 45°C). (B) Boiling dynamics upon (from top to bottom) the Cu, smooth, micro, nano, and 6‐nano‐micro surfaces at the surface superheat of 45°C (Scale bar: 2 mm). (C) Heat transfer coefficient, *h*, within 30 ms for the smooth, micro, nano, and 6‐nano‐micro surfaces at the surface superheat, *T*_s_ *–T*_sat_ (water's saturation temperature), ranging from 45°C to 155°C (*T_sat_* = 100°C). (D) Impact pattern dynamics upon different materials at high temperatures near the LP (Scale bar: 2 mm).

Previous publications have shown that a high *We* (as defined by We=ρvd2Dd/ρvd2Ddσσ [39], where ρ is the water's density, $v_{d}$ is the droplet's impact velocity, $D_{d}$ is the droplet's diameter, and σ is the water's surface tension) enhances droplet boiling heat transfer and delays the LP due to the enhanced convective effects [40]. Therefore, experiments with a high *We* of 274 were first performed as shown in Figure 3. As shown in Figure 3A, when surface temperature reaches 145°C, LP is incipient for the Cu surface and no wetting phenomena were observed during impact as displayed in the first row of Figure 3B. Additionally, droplet trampolining [41] was observed at the LP on the Cu surface. Contrastingly, LP was not observed for droplets upon the silicon‐based surfaces at this value of *We*, as displayed in Figure 3A,B. The smooth and micro surfaces exhibited a tendency to break the break the droplet into multiple Wenzel‐state droplets, which then experienced nucleate boiling as shown in the second and third rows of Figure 3B. As shown in the fourth and fifth rows of Figure 3B, the improved wettability of the nano and 6‐nano structures (see Figure 2) facilitates the formation of a continuous liquid film during boiling, which leads to heat transfer enhancement due to vastly improved liquid–solid contact. On a highly‐wettable surface, liquid–solid contact is analogous to the solid fraction, ψ, which is calculated by ψ=As/AsApAp (where $A_{s}$ is the solid's actual area and $A_{p}$ is the projected area). ψ can be used to characterize a surface's property, with a larger ψ representing a larger liquid–solid contact area and increased number of nucleation sites [42, 43]. ψ for the smooth, micro, nano, and 6‐nano‐micro (as calculated in Section S5.4, Supporting Information) are 1.00, 1.06, 24.6, and 26.1 respectively, indicating that the nano‐fishscale structure fundamentally increases ψ and thus enhances the liquid–solid contact and heat transfer. Correspondingly, the 6‐nano‐micro, obtaining the relatively larger ψ (=26.1) among the five surfaces (Cu, smooth, micro, nano, and 6‐nano‐micro), has the most vigorous nucleate boiling process with the largest temperature drop (19°C in 200 ms), suggesting the proportional relationship between heat transfer capability and the solid fraction and explaining the superiority of the nano‐micro HSS.

As shown in Figure 3C, the heat transfer coefficient, calculated by Equation (1), within 30 ms for the 6‐nano‐micro surface is always the largest for the surface superheat (*T*_s_–*T*_sat_) ranging from 45°C to 155°C, thereby indicating the superior heat transfer capabilities of the hierarchical micro‐/nano‐structures.

(1) $$h=q^{\prime }/\left(T_{s}-T_{d}\right)$$

(2) $$q^{\prime }=Q/A\Delta t$$

where $q^{\prime }$ is the heat flux, obtained by Equation (2), within Δt (30 ms) after the droplet impact, according to Pestana and Head–Gordon's work [44] where the characteristic heat transfer data for a droplet boiling should be obtained within the droplet's impact early stage. Q, calculated in Section S5.1, is the heat transported within Δt (30 ms). A is the area of the target surface (450 mm^2^).

As the superheat exceeds 85°C, the heat transfer coefficient begins to shrink for all tested surfaces. When the surface superheat reached 145°C, an almost‐negligible *h* was detected on the smooth surface, thus indicating the onset of the Leidenfrost regime. The impact dynamics for the smooth surface are illustrated by the representative snapshots in the first row of Figure 3D, where the impinging droplet was broken into smaller droplets that bounced away without any wetting and associated boiling phenomena. Contrastingly, the 6‐nano‐micro surface exhibits a heat transfer coefficient of 1135.4 W/(m^2^·K) for the same conditions, for which the impact and boiling dynamics are shown in the third line of Figure 3D. Although a portion of the impinging droplet is still bounced away from this structure (as can be seen in the frame labelled “5 ms”), the remaining liquid was captured by the hierarchical structure and formed a small but complete liquid film which was seen to boil. As the superheat was increased yet further to 155°C, the temperature drops for all surfaces decreased significantly; indeed, the 242.7 W/(m^2^·K) exhibited by the 6‐nano‐micro surface was still the largest observed drop at this surface temperature. The impact patterns on the micro surface at the superheat of 155°C are presented in the second row of Figure 3D, showing that the bouncing droplets were substantially smaller than at the LP at the superheat of 145°C for the smooth surface. We postulate that the micro‐scaled edges of the micro‐structured surface act to break the impinging droplet into smaller pieces than its smooth analogue, through the provision of lateral bouncing vectors which can more comprehensively scatter the impinging liquid. Similarly, the impact upon the 6‐nano‐micro surface at the superheat of 155°C (bottom of Figure 3D) also shows a bouncing‐away phenomenon without any clear wetting, implying the LP state has been achieved.

**FIGURE 4:**
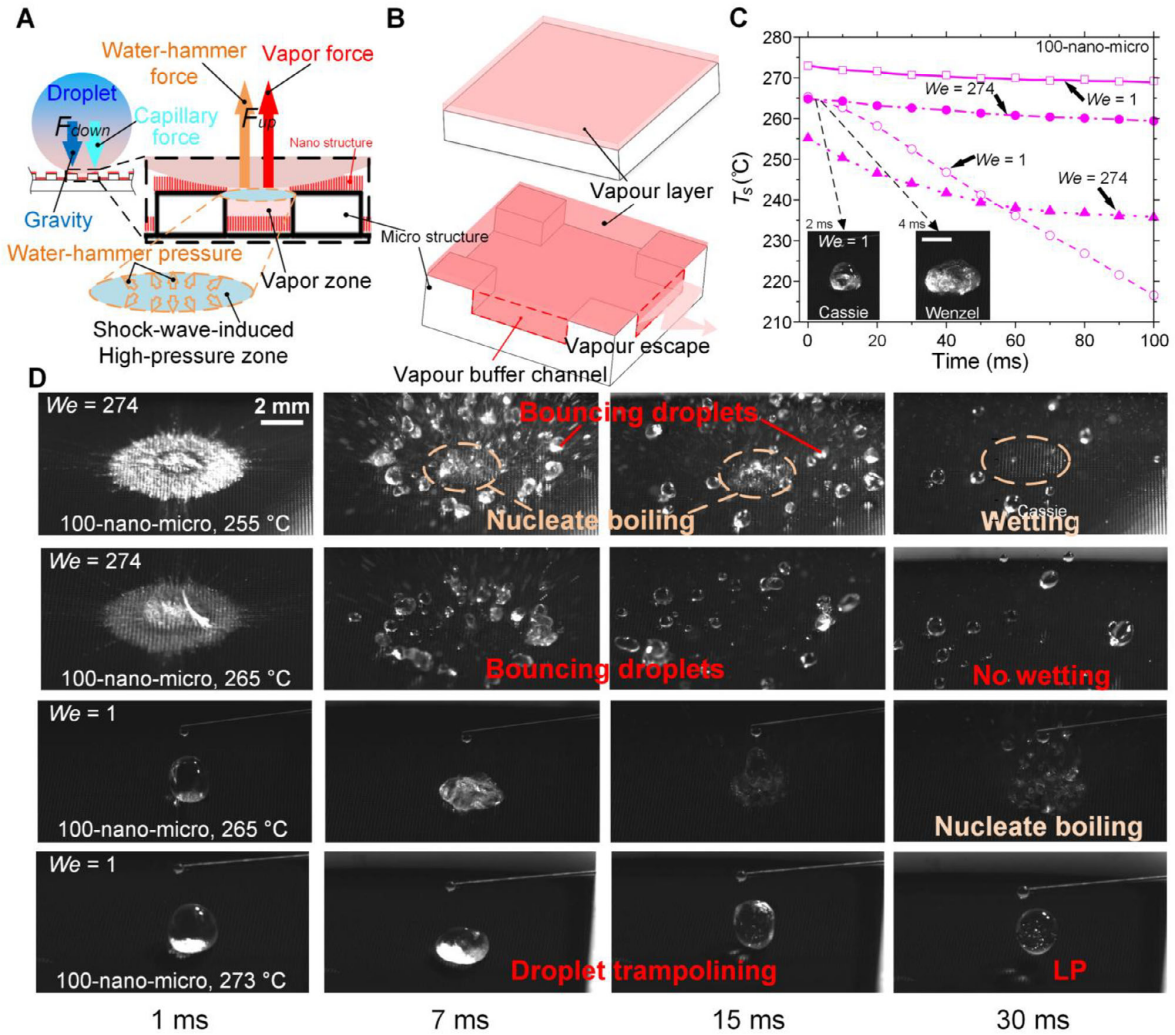
VBE and heat transfer upon the 100‐nano‐micro surface. (A) Force analysis of an impinging droplet in Leidenfrost regime upon solid materials with magnified views of the liquid–vapor–solid interface area and shock‐wave‐induced high‐pressure zone (Schematic not to scale). (B) VBE is caused by the micro‐scale structure (adapted from Wang's work [24]). (C) Temperature trends for droplets impacting upon the heated 100‐nano‐micro surface (≥255°C) with two different Weber numbers (*We* = 1 and 274) (Scale bar: 2 mm). (D) Corresponding impact pattern dynamics for the conditions described in (C).

As was analyzed above, the droplet LP is incipient when the liquid bounces from the surface without wetting. We therefore expect that the key to delaying the LP should be the suppression of the bouncing‐away phenomenon, and it is thus instructive to inspect the causes thereof more closely. As shown in Figure 4A, there are two upward forces $F_{\mathrm{up}}$, which are responsible for the bouncing‐away phenomenon; namely, these are the vapor force $F_{v}$, and water‐hammer force (WHF) $F_{\mathrm{wh}}$. Countering these forces and acting to suppress the bouncing‐away are the droplet gravity G and capillary force $F_{\mathrm{ca}}$, which can both be regarded as downward forces, $F_{\mathrm{down}}$. Therefore, reducing the upward force and/or enhancing the downward force may be feasible ways to delay the LP. $F_{v}=P_{v}A_{v}$ where $A_{v}$ is the force area and $P_{v}$ is the saturated vapor pressure, $P_{v}=f\left(T\right)$, which has a strongly positive correlation with $T_{s}$. In this case, we determine T as $0.5(T_{s}+T_{d})$. When $T_{s}$ reaches 245°C, $P_{v}$ can be on the order of 10^5^ Pa on a smooth or nano surface. Previous work has shown how the micro‐structure can efficiently reduce compressive effects by providing vapor escape pathways [27], and we utilized a similar phenomenon on our own micro‐pillared surface, as illustrated in Figure 4B, the inter‐pillar regions function as vapor buffer channels (VBCs) which buffer and evacuate the generated vapor rapidly and decrease vapor pressure substantially. Quantitatively, the vapor pressure in the micro‐structured surface $P_{v,\mu }$ is calculated as:

(3) Pv,μ=PvVvPvVvVv,μVv,μ,

where $V_{v,\mu }$ is the vapor volume on a smooth or nano surface, and $V_{v,\mu }$ is the calculated vapor volume in a surface with nano–micro hierarchical structures. Refer to Section S5.5 for detailed calculation. The vapor pressure on a 245°C 6‐nano‐micro can be reduced to 1/13$P_{v}$ (≈10^4^ Pa) and thus, the vapor force reduces to 1/13 of its original value, showing the 6‐nano‐micro surface also has VBE while the taller pillars from the 50‐nano‐micro and 100‐nano‐micro surfaces are expected to have sufficient capability to buffer the generated vapor force.

$F_{\mathrm{wh}}$ is caused by a shock‐wave high‐pressure zone, which is highlighted in Figure 4A [45]. $F_{\mathrm{wh}}$ is defined by [45]:

(4) Fwh=Pwh×Awh=αρvsvd×14πDdvdDdvdvsvs2,

where α is the water‐hammer coefficient (taken here as α=0.25 [45]), $v_{s}$ is the local speed of sound, and Ddvd/Ddvdvsvs is the diameter of the shock‐wave high‐pressure zone [45]. For We = 274, where $v_{d}$ equals 2.72 m s^−1^, $P_{\mathrm{wh}}$ also reaches as high as ≈10^6^ Pa. $F_{\mathrm{wh}}$ can be greatly diminished by reducing the droplet velocity, $v_{d}$. For example, as $v_{d}$ is decreased from 2.719 m s^−1^ (We = 274) to 0.166 m s^−1^ (We = 1), $P_{\mathrm{wh}}$ undergoes a hundred‐fold reduction from ≈10^6^ (We = 274) to ≈10^4^ Pa and WHF area $A_{\mathrm{wh}}$ decrease by more than 268 times. Therefore, $F_{\mathrm{wh}}$ undergoes a ≈10^4^ reduction.

$F_{\mathrm{ca}}=P_{\mathrm{ca}}A_{\mathrm{ca}}$ where $A_{\mathrm{ca}}$ is the capillary force area and the capillary pressure $P_{\mathrm{ca}}$ is defined by Pca=2σ/2σll where *l* is the characteristic geometric length (meniscus radius) for utilized surfaces. *l* for the HSS is 3 mm and $P_{\mathrm{ca}}$ for the HSS is in an order of 10^1^ Pa (Refer Section S5.6 for detailed calculation). Another downward force G can be regarded as the gravity of the droplet. As analyzed above, The VBE and decreased $v_{d}$ fundamentally lower the upward forces $F_{v}$ and $F_{\mathrm{wh}}$, significantly decreasing the net upward force, defined by Equation (5). Such effects potentially delay the LP.

(5) $$F_{↑}=F_{v}+F_{\mathrm{wh}}-F_{\mathrm{ca}}-G$$

For experimental validation, we observed the impact of a low‐*We* droplet upon a nano‐micro HSS, which has a high aspect ratio micro‐structure (named 100‐nano‐micro). It has the largest ψ of 49.3. In Figure 4C, we plot the surface temperature trends upon the 100‐nano‐micro at high surface temperatures. We chose to use surface temperatures, $T_{s}$ ≥ 255°C, because LP was observed upon the 255°C 6‐nano‐micro surface for We = 274 and 1 (See Figure 3C). A clear temperature decrease is observed for the 255°C 100‐nano‐micro surface with We = 274 (10.8°C decrease within 30 ms), indicating that the LP is significantly delayed when compared with the 255°C 6‐nano‐micro surface. This observation validates the theorized ability of VBCs to delay the LP. However, when the elapsed time exceeds 60 ms, the surface is cooled relatively slowly, which indicates drying out of the surface. Snapshots from footage of the corresponding impingement and wetting processes are shown in the first row of Figure 4D (Video S1). We observed that, in the first 15 ms, there are a large number of bouncing droplets that bounce off the surface due to, which explains the dry‐out in the late phase) although there is visible nucleate boiling at the core of the impact. A liquid film is still observed at 30 ms, which is responsible for the efficient heat transfer. Returning to Figure 4C, we see that very little cooling is achieved when $T_{s}$ raised to 265°C, with the same We, implying that the LP has been reached. We verify this in Figure 4D’s second row, wherein no wetting can be seen upon the 265°C 100‐nano‐micro surface; the VBCs alone are visibly insufficient to further delay the LP. Contrastingly, we see that by lowering $v_{d}$ (We = 1) to decrease $P_{\mathrm{cwh}}$, we can again delay the LP on the 265°C 100‐nano‐micro surface. As shown in Figure 4C, the surface temperature reduces rapidly and consistently from 265°C. Although the rate of cooling for this condition is initially (within 10 ms) smaller than that of the 255°C 100‐nano‐micro surface with We = 274, the temperature reduction grows continuously, thus suggesting a persistent heat transfer enhancement. High‐speed footage of the early stages of droplet boiling (2 and 4 ms), inset in Figure 4C, displays a transition from the Cassie to Wenzel state, which allows greater droplet‐solid contact and subsequent boiling processes as shown in the third row of Figure 4D (Video S2). There are no obvious $P_{\mathrm{cwh}}$‐induced bouncing droplets in the early stages of droplet‐solid contact (before 7 ms). Although a certain number of droplets do eject away, caused by, possibly, violent vapor force, can be observed when the vigorous nucleate boiling happens at the time of 30 ms, the total amount of ejected liquid is much smaller than that in the first row of Figure 4D which caused by both $P_{\mathrm{cwh}}$ and $P_{v}$, which can explain the persistence of the cooling process in this condition. Finally, we observed in Figure 4C that when $T_{s}$ of the 100‐nano‐micro is raised to 273°C, the LP is incipient even for We = 1. Snapshots of the high‐speed footage for these conditions are shown in the bottom row of Figure 4D, verifying that the LP has been reached as droplet trampolining occurs (Video S3).

**FIGURE 5:**
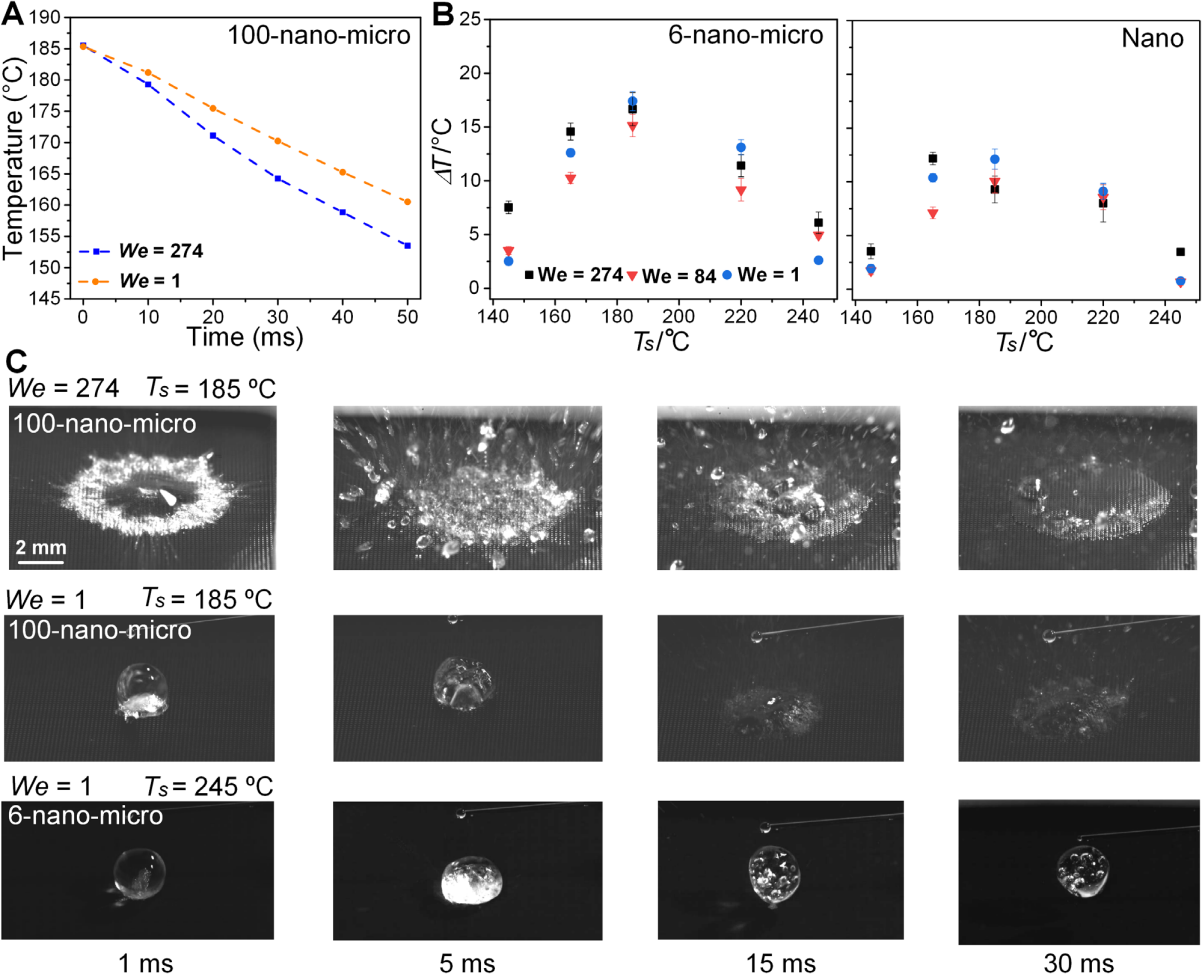
Weber effect on the heat transfer performance under various conditions. (A) Temperature trends during droplet impact (*We* = 1 and 274) at surface temperature of 145°C 100‐nano‐micro. (B) Temperature drops, Δ*T*, after 30 ms for surfaces of 6‐nano‐micro and nano at surface temperatures, *T_s_*. ranging from 145°C to 245°C and various *We*. (C) Impact pattern dynamics of typical conditions for the 100‐nano‐micro and 6‐nano‐micro surfaces.

However, a low We does not persistently enhance the droplet boiling as shown in Figure 5 due to a competition between convection and phase‐change heat transfer. Figure 5 presents Weber effect on the heat transfer performance for wider operating conditions. As shown in Figure 5A, the temperature drop rate from a low‐*We* impacting droplet upon 185°C 100‐nano‐micro is inferior to that from a high‐*We* droplet, indicating the heat transfer enhancement is dominated by the strong convection effect. The first row of Figure 5C shows the strong splash and wetting phenomena for the 100‐nano‐micro surface, caused by the high‐*We* droplet. In contrast, the impact pattern of the low‐*We* droplet, shown in the second row of Figure 5C, is much weaker than those above, explaining the reduced heat transfer capability. It indicates that a high *We* is preferred in the nucleate boiling region, which is far away from the LP, which maximizes the convection heat transfer. *We* effect of the 6‐nano‐micro and nano surfaces is plotted in Figure 5B. A higher *We* boosts heat transfer performance when *T*_s_ is under 165°C or equal to 245°C. In the low‐*T*_s_ area, a strong convection during the droplet impact triggers heat transfer enhancement. The coupling effect of convection and phase change results in an enlarged heat transfer ability. In the 245°C *T_s_* case, the high kinetic energy of the impact droplet helps a small amount of liquid to weakly penetrate the dense vapor layer, thus wetting the surface and improving the heat transfer, as shown in the third row of Figure 3D. However, this effect cannot be obtained in the last row of Figure 5C with a low *We*, leading to a complete LP. The insufficient vapor buffer effect and weakened kinetic energy explain the reduced heat transfer for both 6‐nano‐micro and nano surface in the 245°C *T_s_* cases. The superiority of the low‐*We* effect in the high *T*_s_ case can only be triggered by a sufficient vapor buffer effects.

**FIGURE 6:**
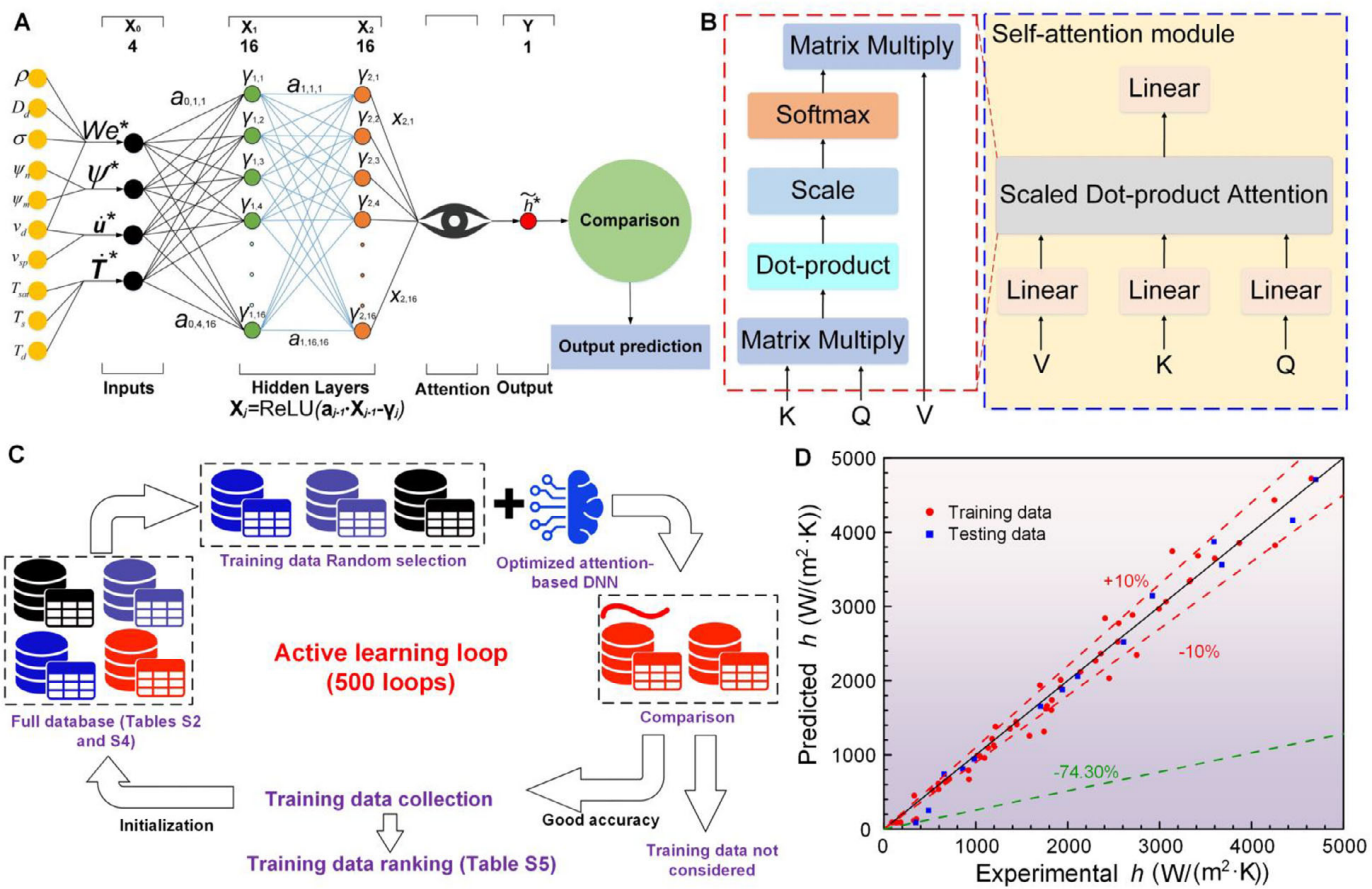
Physics‐informed attention‐integrated DNN model integrated with a self‐attention model and prediction results. (A) Topological structure of the DNN model. The physics‐informed model comes from the input parameters $X_{0}$ are obtained from original data processing with physics knowledge. The original data resides in the first column. Through data processing, $X_{0}$ is composed of four dimensionless parameters that have physical meaning. (B) Schematics of the self‐attention module. (C) A schematic view of the active learning algorithm. (D) Prediction results in both the training and texting dataset.

In general, the droplet boiling heat transfer behavior has a complicated and nonlinear relationship among multiple critical parameters such as surface temperature, Weber number, wettability, VBE, and liquid–solid contact intensity. Here, we adopt h as a dependent variable to characterize the heat transfer behavior. A model, which is presented by Equation (6), is established to predict h. Traditionally, power‐law equations were extensively adopted in heat transfer and fluid flow modeling, but they lack universality. Here, we utilize a data‐driven physics‐informed attention‐integrated DNN model (refer Section S6) integrated with a self‐attention module [46] (see Methods for the model establishment) to accurately model h based on all the heat transfer data listed in Table S4. Figure 6A shows the optimized structure of the attention‐integrated DNN model [47]. Figure 6B demonstrates the mechanism of the attention mode. The optimized hyperparameters (learning rate, epoch, and batch size) are 0.004, 2000, and 64, respectively. The structure and hyperparameter optimization processes are subject to our recent work [48]. An active learning algorithm is adopted for selecting optimal training data for the purpose of small‐sampling DNN modeling [49] without sacrificing prediction precision. The active learning algorithm used in this study is illustrated in Figure 6C. We conducted five hundred times [48] of the model, referred to as the five hundred active learning loops, using the optimized attention‐integrated DNN [50]. For each iteration, the training data was randomly selected, maintaining a training‐to‐testing data ratio of 4:1. The prediction results varied for each loop due to the random selection of training data. If the error rate (calculated in the testing dataset) was 10% or less, the corresponding training dataset was preserved. After completing the five hundred loops, we recorded the frequency of each data point in the preserved training datasets. The results, sorted in descending order, are presented in Table S5. We also examined the impact of varying the number of training data and selected different quantities for prediction using the optimized attention‐integrated DNN. For these training datasets, data points with higher frequencies were prioritized, as they demonstrated a higher potential for achieving accurate predictions. Based on Section S8, we select 53 data points as the optimal training dataset. Figure 6D plots the best prediction results among the 100 runs of the attention‐integrated DNN with 53 optimized training data. It demonstrates the detailed comparisons between predicted and experimental results in both the training and testing datasets, where most predicted data points reside within the ±10% relative error lines. The average relative error in the testing dataset is 12.9%. It can be seen that one prediction's relative error in the testing dataset is relatively large (−71.3%), which increases the average relative error there. Such a large error is caused by the small experimental *h*’s absolute value (because it is in the Leidenfrost region). It can be decreased from 12.9% to 8.2% when not considering the data point with the largest error. The overall average relative error is only 11.8% in both the training and testing datasets. The model validation using the heat transfer data using the 50‐nano‐micro is displayed in Section S9. The results indicate that the established model has learned the nonlinear heat transfer laws of the droplet boiling upon solids with different temperature regions, wettability, and VBCs.

(6) $$h=f(T^{\prime },We,\psi ,u^{\prime })$$

where $T^{\prime }$ is the dimensionless surface temperature and $u^{\prime }$ is the dimensionless spreading velocity. Refer to Sections S4.2 and S4.3 for parameter calculation.

## 3 Discussion and Conclusion

**FIGURE 7:**
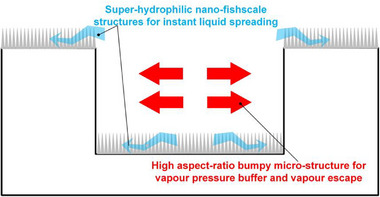
Operational mechanisms of the nature‐inspired nano‐micro hierarchical triple‐passage architecture for sustained boiling. On top and bottom of the periodic bumpy micro‐pillars, the nano‐fishscale structure spreads the liquid rapidly, functioning as two liquid delivery passages. Micro‐grooves between the top and bottom nano‐structures function as a vapor escape passage. Schematic not to scale.

Although nano‐micro hierarchical structures have been proved effective in augmenting critical heat flux in the pool boiling [43, 51, 52, 53], their applications in the droplet boiling area for LP delay have not been comprehensively reported. Inspired by natural super‐hydrophilic structures, we designed and fabricated tailor‐made nano‐micro hierarchical structures for sustained and enhanced droplet boiling heat transfer due to a triple‐passage architecture design, shown in Figure 7, where vapor and liquid spreading dynamics are both enhanced. The nano‐structure provides abundant nucleation sites, whereas the micro‐structure offers a vapor buffer effect, both of which improve liquid–solid contact and heat transfer efficiency. Both high‐*We* and low‐*We* droplets were utilized to analyze and compare the impact velocity effect on the boiling performance. Our results challenge the conventional belief that a higher impact velocity necessarily results in improved phase‐change heat transfer. This is due to the increased vapor force and WHF, which dominantly promote the occurrence of LP and cause a rapid deterioration in heat transfer properties on a relatively high‐temperature solid. Our experiments revealed enhanced heat transfer performance in the low‐*We* regime at large superheats, attributed to the significantly reduced WHF. However, such low‐*We*‐induced heat transfer improvement can only be triggered by nano‐micro HSSs with spacious VBCs. The spray cooling technique, which involves the collective impact‐boiling of numerous high‐velocity droplets [54, 55], has been widely used as an effective heat dissipation method [56]. However, the findings here suggest that employing a high‐*We* droplet for boiling on a relatively high‐temperature solid may not always be an ideal cooling solution. Conversely, a very low droplet velocity (and correspondingly low *We*) fundamentally decreases WHF, thereby promoting wetting enhancement at the expense of convective cooling effects.

Although promising, limitations exist here. As listed in Tables S9 and S10 (Section S10), the obtained heat transfer performances, including LP temperature and heat transfer coefficient in this paper, are not sufficiently excellent compared to the latest publications. Although the purpose of this paper is not to chase a new record in a higher surface temperature, the authors admit this limitation. Furthermore, the established DNN model is specifically calibrated for distilled water as the coolant, and the four input parameters of the DNN have been defined with applicable ranges. *We* should fall within the range of 1.00 to 274; ψ should be within the range of 1.00 to 49.3; $u^{\prime }$ should be within the range of 2.200 to 162.2; and $T^{\prime }$ should be within the range of 0.6 to 2.2.

In summary, this paper investigates impinging droplet boiling processes on seven solid surface designs, conducting theoretical and experimental analyses to identify factors that enhance heat transfer and suppress the Leidenfrost incipience. By designing nature‐inspired nano‐micro HSS (which possesses triple‐passages for liquid and vapor delivery) and controlling impinging droplet characteristics, we extend the nucleate boiling regime by nearly 130°C. Enhanced liquid spreading dynamics and high surface solid fraction, facilitated by the nano‐fishscale structure, promote rapid liquid‐solid contact, enabling efficient heat removal. High‐aspect‐ratio structures create spacious VBC, where the vapor force can be significantly dampened beneath the droplet, and low *We* droplets diminish the water‐hammer force, both of which synergistically delay the occurrence of Leidenfrost. We established a multi‐forces competition model to quantitatively show such Leidenfrost delay and developed a physics‐informed DNN model that accurately reflects these synergistic effects across a wide range of temperatures and various HSS configurations. Our novel insights shed light on the design and control of more efficient droplet‐based phase‐change heat transfer devices.

## 4 Methods

### 4.1 Materials and Surface Fabrication

Except the Cu surface, the other six surfaces were fabricated on 525 µm‐thick, single‐side polished, n‐type silicon wafers in class 1000 and class 100 cleanrooms. All the micro and nano‐micro surfaces were fabricated by standard photolithography and etching processes. The smooth surfaces were fabricated by depositing a 200 nm‐thick SiO_2_ film on the wafer using low‐pressure chemical vapor deposition. All of the nano‐structured surfaces (namely the nano, 6‐nano‐micro, 50‐nano‐mocro, and 100‐nano‐micro surfaces) were created by performing inductively‐coupled plasma deep reactive‐ion etching processes on both unstructured and micro‐structured silicon wafers. Detailed surface fabrication steps are provided in Section S4. Before performing experiments, all surfaces except the Cu surface were treated with O_2_ plasma to grow a thin layer of hydrophilic hydroxy groups, thus hydrophilizing the surfaces.

### 4.2 Experimental Methods

The temperature data was collected by NI 9213 and NI cDAQ‐9184 (National Instruments Co., Ltd.), whose sampling rate was set to be 100. The experimental study was conducted with the following conditions: for generating a droplet with *We* of 84 and 274, the syringe pump was employed, while a manual operation was utilized for *We* = 1. In the manual operation, the operator carefully positioned the needle head at a fixed distance of 2 mm above the target surfaces. The SEM images were acquired using a Thermal Field Emission Scanning Electron Microscope (GeminiSEM 300) from Carl Zeiss AG. The 3D morphology image presented in Figure 1D was obtained utilizing a 3D Measurement Laser Microscope (LSM 700) from Carl Zeiss AG.

### 4.3 Physics‐Informed DNN Modeling Method

Compared with the traditional DNN model [57, 58], the attention‐integrated DNN, proposed by Vaswani et al. [46], is a newly developed deep learning algorithm, integrating both traditional DNN and attention modules. The final output of the physics‐informed attention‐integrated DNN is calculated by:

(7) $$\tilde{y}={\tilde{h}}^{*}=\mathrm{Attention}(X_{L}),$$

where $X_{L}$, calculated by Equation (8), is the output of the last hidden layer and input matrix of the self‐attention module. As shown in Figure 6A, the subscript *L* equals two as there are two hidden layers in the optimized structure of the physics‐informed attention‐integrated DNN model.$\mathrm{Attention}(X_{L})$ denotes the exertion from the attention module, which locates in the downstream of the hidden layer. The detailed operation mechanism of the attention module is shown in Figure 6B. Please refer to Vaswani et al.’s work [46] for the detailed calculation method and code of this established attention‐integrated deep learning algorithm.

(8) $$X_{L}=Re\mathrm{LU}\left(A_{j-1}X_{j-1}+\gamma_{j-1}\right)\left(j=1,2,\text{…},L\right)$$

where is the bias matrix of the (*j *− 1)th hidden layer, $A_{j-1}$ is the weight matrix of the (*j*‐1)th hidden layer, $X_{j-1}$ is the input matrix of the (*j* − 1)th hidden layer. $X_{0}$, defined by Equation (9), is the vector composed of the four normalized input variables (The method of normalization is presented in Equation (10)). The ReLU is a symbol of Rectified Linear Unit, a non‐linear transfer function [59, 60], which is adopted in the hidden layers of the deep learning framework as the activation function.

(9) $$X_{0}=\left[x_{0,1},x_{0,2},x_{0,3},x_{0,4}\right]=\left[We^{*},\psi^{*},{u^{\prime }}^{*},{T^{\prime }}^{*}\right]$$

(10) $$x^{*}=\frac{x-x_{\min}}{x_{\max}-x_{\min}}=\left(x=We,\psi ,u^{\prime },T^{\prime }\right)$$

where, $x_{\min}$ and $x_{\min}$ are the minimum and maximum values for each variable, respectively.

## Conflicts of Interest

The authors declare no conflicts of interest.

## Supporting information




**Supporting Information file 1**: exp270075‐supp‐0001‐SuppMat.pdf

**Supporting Information file 2**: exp270075‐supp‐0002‐SuppMat.zip

## Data Availability

The data that support the findings of this study are available from the corresponding author upon reasonable request.
