# Supplementary material for: Sustained and Enhanced Nucleate Boiling Using Hierarchical Architectures at Large Superheats
Source: Exploration (Beijing). 2025 Aug 25;5(5):20240137. doi: 10.1002/EXP.20240137 (PMC12561293; doi:10.1002/EXP.20240137)
Supplement: Supplementary file 1 — Supporting Information file 1: exp270075‐supp‐0001‐SuppMat.pdf [file EXP2-5-20240137-s002.pdf]

Supporting Information for

Sustained and Enhanced Nucleate Boiling using Hierarchical Architectures at Large Superheats

Ji-Xiang Wang, Hongmei Wang, Christopher Salmean, Binbin Cui, Ming-Liang Zhong, Yufeng Mao, Jia-Xin Li, Shuhuai Yao

This PDF file includes:

Supplementary Text
Figures. S1 to S12
Tables S1 to S10
Videos S1 to S3
References ([1] to [16])

S1 Detailed description of the experimental set-up

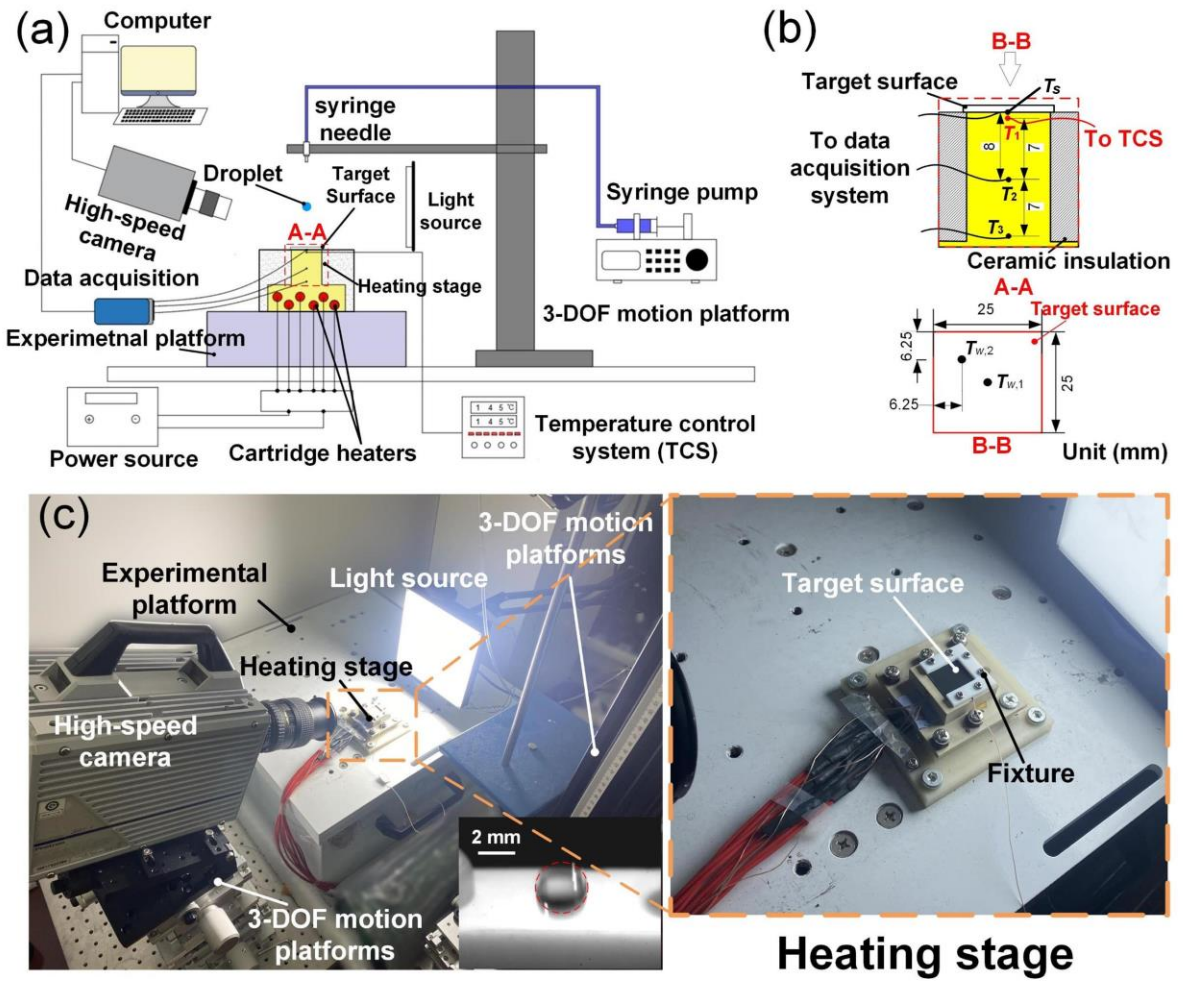

Figure S1. Experimental system. (a) A detailed schematic view and (c) digital image of the experimental system. (b) Cross-sectional view and top view of the target surface area. The insert in (c) show a falling droplet captured by the high-speed camera. The magnified view in (c) shows the photographic view of the heating stage.

As shown in **Figure S1**, a 27G syringe needle is installed in a three-DOF motion platform, which conveniently regulates the droplet impact location upon the center of the target surface. Room-temperature droplets ($T_d = 20$ ℃) were produced from above using a 27G syringe needle ($D_d = 2.7$ mm) and a syringe pump (LSP01-1BH, Longer Pump Co., P.R. China). A copper-based heating stage, highlighted in **Figure S1 (a)**, with a 2 cm × 2 cm square head was fixed to the vibration stage. Six 200W cartridge heaters, brought from Jiangsu Xinghe Co., Ltd., were inserted into the heating stage. The six heaters were connected to a power source (N5766), brought from Keysight Co., Ltd., to generate heat load. The heating stage was wrapped with a layer of Teflon to reduce heat loss. A target surface can be fixed to the square head with silicon grease material (Arctic Silver 5) using

screws, which can conveniently replace surfaces after each run of the experiment. An enlarged cross-sectional view of A-A surface is displayed in **Figure S1 (b)** where four temperature measurement points (T_s, T_1, T_2, and T_3) were located into and on the head area of the heating stage to measure the temperature information at key locations. T_w, obtained by $T_s = 0.5\left(T_{s,1} + T_{s,2}\right)$, recorded the average temperature at the interface between the heating stage head and target surface wall. $T_{s,1}$ and $T_{s,2}$ are obtained by two thermocouples upon the head of heating stage. The detailed locations of these two thermocouples are presented in the B-B plane shown in **Figure S1 (b)**. T_1, T_2, and T_3 recorded temperatures along the heating stage head horizontally to calculate the heat flux. The distances d_{12} (between T_1 and T_2) and d_{23} (between T_2 and T_3) were 7 mm. The distance between T_1 and the heating stage surface d_{1w} was 1 mm. The thermocouple, which measures T_1, was connected to a temperature control system (TCS), whose operating mechanism is described in the following paragraph, to regulate the power source to have T_1 controlled and thus T_s can be controlled as well. The other three temperatures (T_s, T_2, and T_3) were recorded by the data acquisition system (NI 9213 from National Instruments Co., Ltd.), whose sampling rate was set to 100. It means the system could collect temperature every 10 ms. A high-speed camera (Photron Fastcam SA2), as shown in **Figure S1 (c)**, with a light source was installed to observe the droplet boiling pattern. The capturing frequency of the high-speed camera was set to 2000 1/s. Thermocouples, produced by Omega Co., Ltd., used in this study are unsheathed thermocouples with the diameter of 0.5 mm. The thermocouple's accuracy is ± 0.1 ℃.

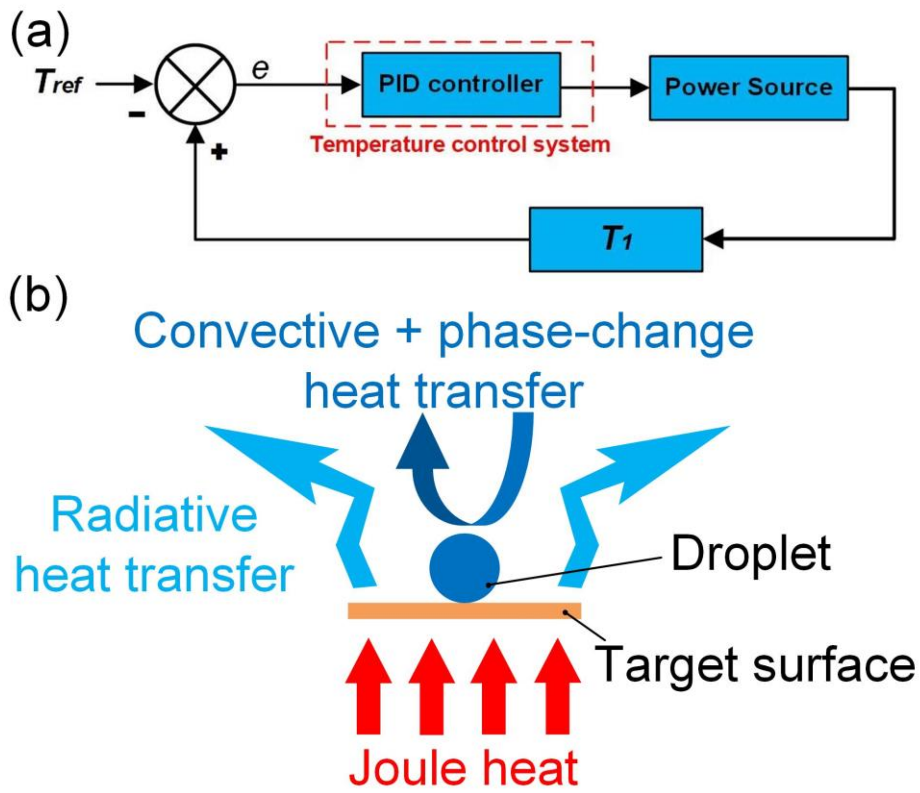

Figure S2. Temperature control and boundary effect of the target surface. (a) Operating mechanics of the temperature control system. (b) Boundary effect of the target surface.

The temperature T_1 was controlled through a TCS, which is driven by a PID controller as shown in **Figure S2 (a)**. The PID controller in the TCS can control the on/off the power source by comparing the real-time T_1 and the T_{ref}. When T_1 is smaller than T_{ref}, the controller powers on the power source and when T_1 is larger than T_{ref}, the power source is powered off, so the heating system and surface's temperature can be controlled. As demonstrated in in **Figure S2 (b),** the PID controller controls the Joule heat that conducts into the backside of the target surface, which elevates the surface's temperature. On the other side of the target surface where the droplet hits, convective and phase-change heat transfer takes place to cool the surface. Additionally, radiative heat transfer takes part in the heat dissipation considering the high temperature of the surface. The radiative heat transfer is calculated by $Q_{ra} = \varepsilon \omega A\left(T_s^4 - T_e^4\right)$ where ε is emissivity of the surface (~ 0.9 for etched nanostructured black silicon wafer), ω is the Stefan-Boltzmann constant (5.67 $\times 10^{-8}$ W·m^{-2}·K^{-4}), A is the surface area, T_s is the surface temperature (418.15 ~ 546.15 K), and T_e is the ambient temperature (293.15 K). Therefore, Q_{ra} is in the range between 0.53 to 1.87 W. It can be concluded that

the radiative heat transfer rate constitutes only a minor fraction of the total dissipated heat transfer rate. The convective and phase-change heat transfer through the droplet, even within the Leidenfrost regime, is on the order of 10^1 W (with the highest heat transfer rates in the nucleate boiling region exceeding 300 W).

In the experiment, it was necessary to ensure that the high-speed camera captured droplet boiling patterns were consistent with the recorded temperature in time, thus a software called “multi position simultaneous clicker for mouse” was applied to initiate the data recording and high-speed camera capture at the same moment. The software interface of the “multi position simultaneous clicker of mouse” is shown in **Figure S3**. The software can record the two mouse clicking locations’ coordinate information in advance. These two mouse clicking locations are these two buttons to initiate the data recording software (located in the left-side of **Figure S3**) and the high-speed camera software (located in the right-side of **Figure S3**). When initiate the “multi position simultaneous clicker of mouse” software, it can click the two positions synchronously. In this way, when the high-speed camera starts to capture droplet boiling behaviour, the temperature acquisition system also starts to work. After each run of experiment, we can obtain the dynamic droplet pattern alone with the corresponding time-synchronized temperature changes. In each run of experiment, we regard t = 0 when the droplet first contacts the solid wall.

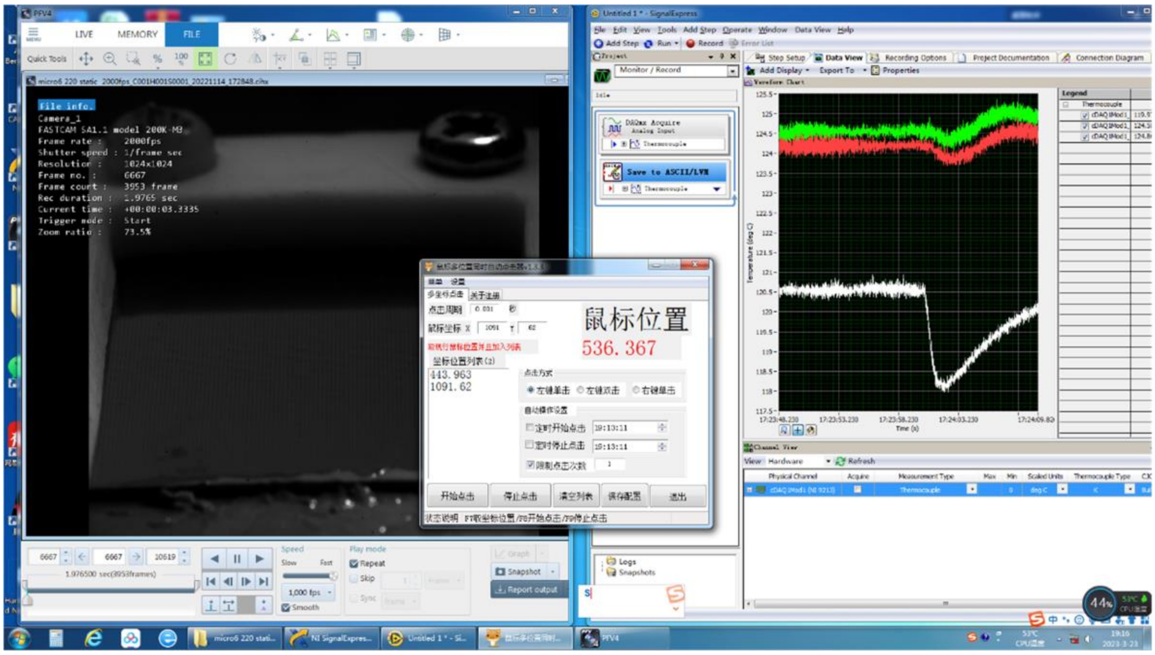

Figure S3. Image of the software interface to initiate the temperature data collection software and high-speed camera capturing software synchronously.

S2 Wettability test of the Cu, micro, and nano surfaces

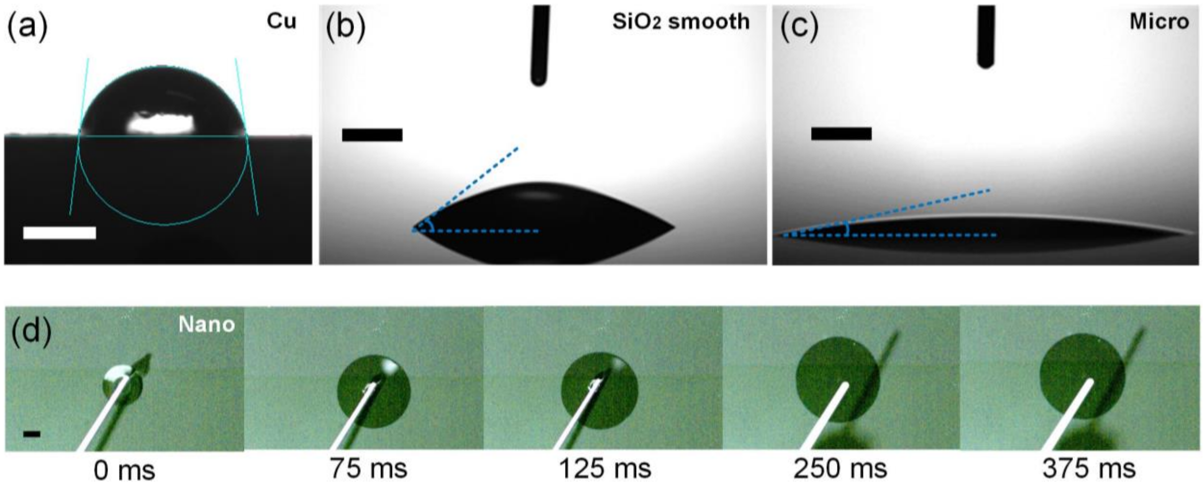

Figure S4. Wettability characterization of the Cu, micro, and nano surfaces (Scale bar: 1 mm). Contact angles of (a) Cu, (b) smooth, and (c) micro surfaces. (d) Wetting dynamic images of the nano surface.

S3 Reasons for designing and fabricating micro-pillared surfaces

S3.1 Nature inspiration

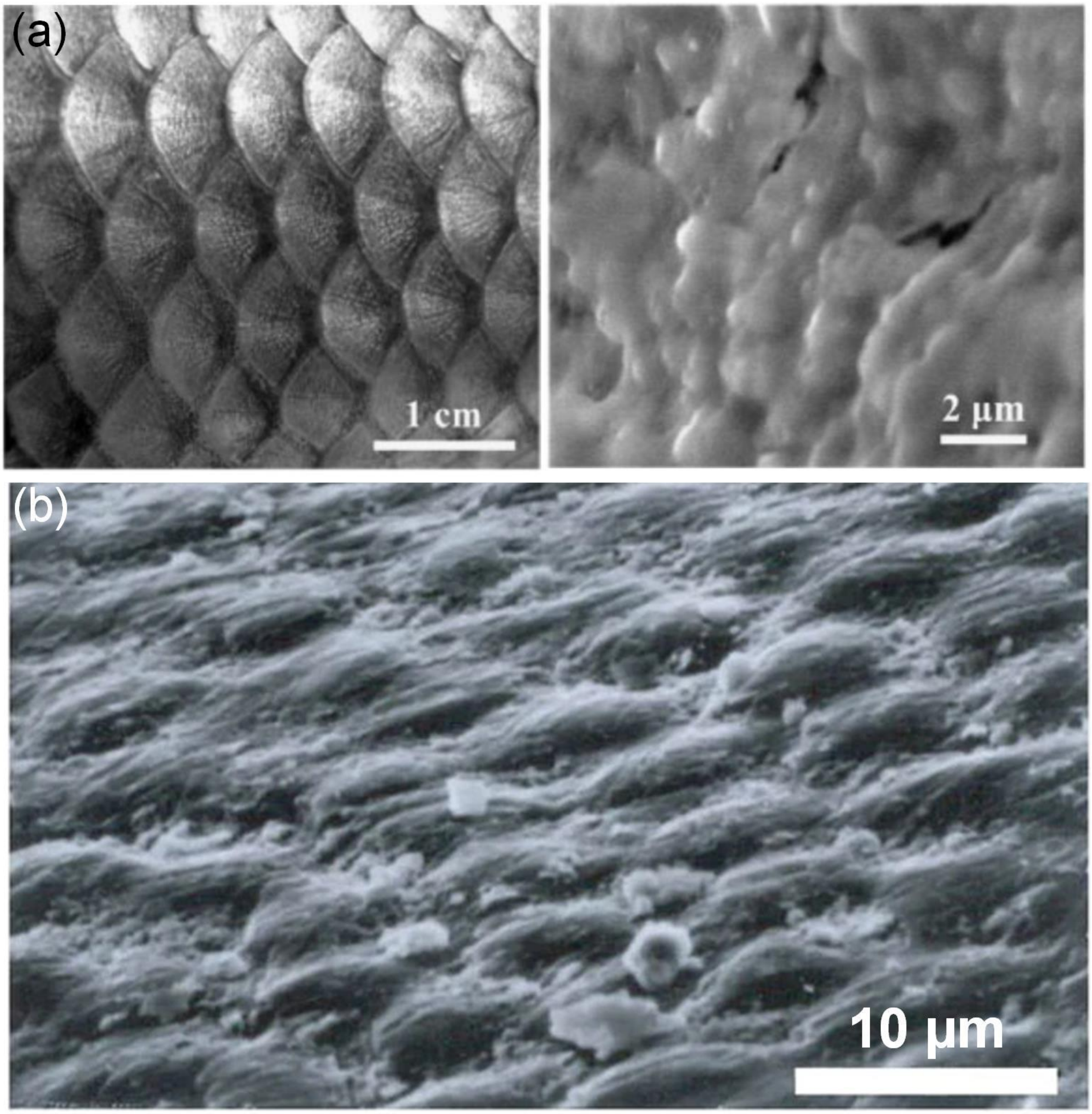

Figure S5. Material design inspired by nature biological organisms in this paper. (a) Superhydrophilic fish scale [1]. (b) Desert beetle's bumpy structure [2].

S3.2 Engineering purposes for enhanced heat transfer

This study employs micro and nano solid materials that are based on a surface patterned with bumpy micro-pillars. Besides the biomimetic design principles shown in Section S3.1, this particular pattern is a common micro-structured morphology that is used to improve boiling heat transfer [3]. The use of

a micro-pillar-patterned surface is favoured due to its relatively straightforward manufacturing process, efficient solid-liquid interaction, and high solid fraction. The solid fraction is a measure that indicates the ratio of the actual wetting area to the projected area. Other morphologies, such as micro-pyramids, are capable of easily propelling droplets due to the Laplace pressure [4]. However, this characteristic makes them less appropriate for boiling applications and more suitable for condensation surfaces. The design of the 100 μm × 100 μm micro-pillars in this research is influenced by the spray cooling work of Chen et al. [5], where a 26% increase in heat flux was achieved compared to a smooth reference surface.

S4 Surfaces fabrication and characterization

S4.1 Surface fabrication

Except from the Cu surface, the other six surfaces were fabricated on 525-μm-thick single-side polished N-type silicon wafer, in the class 1000 and class 100 clean rooms of HKUST's Nanosystem Fabrication Facility (NFF-CWB). The wafers were immersed in piranha (H2SO4:H2O2:H2O) solution at 120 °C for 10 min, followed by SiO_2 removal with room-temperature HF solution for 1 min. The wafers were then dump-rinsed with deionized (DI) water and spin-dried with nitrogen gas. The smooth surfaces were fabricated by depositing a 200 nm-thick SiO_2 film on the wafer using low-pressure chemical vapour deposition.

All the micro and nano-micro surfaces were fabricated by standard photolithography and etching processes. As shown in **Figure S6**, the fabrication of bumpy micro-pillar arrays followed the standard photolithography. Positive photoresist (HPR506, OCG Microelectronic Materials, Belgium) was spin-coated on the thoroughly cleaned Si wafer at 2 krpm (thickness ~3 μm) and soft-baked on a hotplate at 110 ℃ for 60 s with the SVG88 coater track (Rite Track, USA). Next, the photoresist coated wafers were exposed to 237.5 mJ/cm^2 UV through a 5-inch Cr/quartz photomask, which defined the pattern of the micro-array, using the SUSS MA-6 mask aligner (SUSS MicroTec, Germany), then puddle-developed using tetramethylammonium hydroxide (TMAH)-based developer (FHD-5, Fujifilm, Japan) for 60 s in the SVG88 developer track. After hard-bake in 120 ℃ oven for 30 min, the patterned silicon wafer was dry-etched using deep reactive-ion etching (DRIE) (Omega Rapier XE, SPTS, UK). The cyclic etching and passivation steps in DRIE allow highly anisotropic and selective etching into the wafer and subsequent formation of uniform and perpendicular pillars, with the pillar height controlled by the number of alternating etching and passivation cycles. Finally, the photoresist was then removed from the wafers using oxygen plasma.

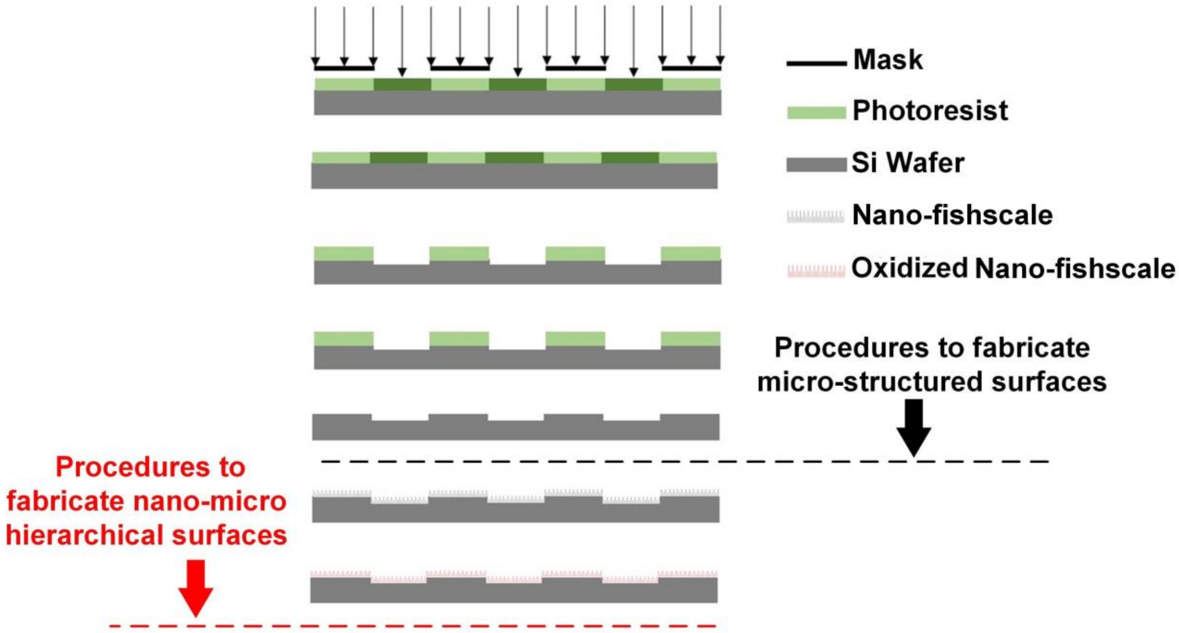

Figure S6. Procedures to fabricate utilized nano- / micro-structured surfaces.

All of the nano-structured surfaces (namely the nano, 6-nano-micro, 50-nano-micro, and 100-nano-micro surfaces) were created by performing inductively-coupled plasma (ICP) DRIE processes on both unstructured and micro-structured silicon wafers. As shown in the procedures below the black dotted line in **Figure S6**, subsequent ICP DRIE steps (Surface Technology Systems, United Kinddom) were carried out on micro-structured wafers in order to create the nano-grass features of the nano-micro hierarchical structured surface (HSS). The surfaces were stripped of all silicon dioxide using buffered HF solution, and then the Black Silicon method was employed to create nano-fishscale on the unmasked surfaces. The ICP DRIE contains periodic passivation and etching processes where SF_6 and C_4F_8 were utilized as reactants. The detailed parameters for the ICP DRIE are described as follows: the coil power was regulated to 550 W, the chamber pressure was set to be 25 Torr and 293 K. For the passivation cycle, the flow rate of C_4F_8 was around 80 standard cubic centimeter per minute (sccm). For the etching cycle, the flow rate of SF_6 was approximately 70 sccm. The platen power was regulated to be about 15 W. After that, uniform nano-structures were consequently yielded, with respective heights of around 2.89 μm. By applying this technique to the micro-structured silicon wafer, nano-micro HSSs could be fabricated. After thoroughly cleaning, all wafers were treated with O2 plasma to grow a thin layer of silicon dioxide on their sidewalls and other etched areas, thus hydrophilizing the wafers.

S4.2 Cu surface characterization

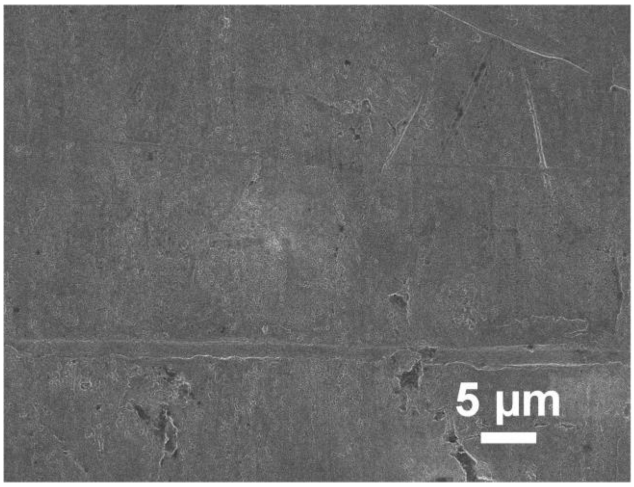

Figure S7. SEM images of the utilized Cu surfaces.

Figure S7 displays the SEM image of the utilized Cu surface.

S5 Parameters determination

S5.1 heat transfer coefficient calculation

The heat transfer coefficient *h* is calculated by Eq. (1). In Eq. (1), Q is defined by:

$$Q = c_{si} m_s \Delta T \,, \tag{S1}$$

where c_{si} is the specific heat capacity of the silicon material, m_s is the mass of the target surface (listed in **Table S1**). ΔT is the temperature drop within 30 ms.

Table S1. Masses of the different surfaces.

Surfaces	Smooth	Micro	Nano	6-nano-micro	50-nano-micro	100-nano-micro
Mass (g)	0.772	0.750	0.756	0.745	0.711	0.657

S5.2 Dimensionless surface temperature calculation

The initial surface temperature T_s matters a lot on the heat transfer. Therefore, a dimensionless surface temperature is obtained by:

$$\dot{T} = \left(T_s - T_{sat}\right) / \left(T_{sat} - T_d\right), \tag{S2}$$

where T_{sa} is the saturation temperature of the water under atmospheric pressure.

S5.3 Dimensionless spreading velocity calculation

The dimensionless spreading velocity is defined as the ratio of droplet's impact velocity v_d to the liquid spreading velocity v_{sp} within 50 ms upon the specific surfaces, as presented in Eq. (S3). **Table S2** lists v_d with different *We*.

$$\dot{u} = v_d / v_{sp} \tag{S3}$$

Table S2. v_d for different *We*.

We	1.00	84.0	274
v_d (m/s)	0.16	1.5	2.7

S5.4 Solid fraction calculation

ψ for the smooth should be 1. The determination of ψ *fo*r the micro-structure is determined with the assistance of **Figure S8**. It can be obtained by [6]:

$$\psi_m = \left(a^2 + 4ah_\mu\right) \big/ p_\mu^2 , \quad \text{(S7)}$$

where a is the length and width of the micro-pillar (100 μm), h_μ is the height of the micro-pillar (6.4 and 100 μm), and p_μ is the width and length of the unit cell (200 μm).

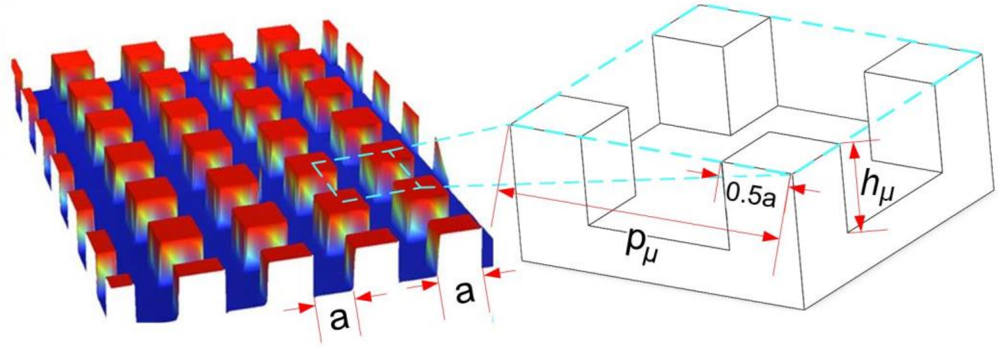

Figure S8. 3D morphology of the micro-pillars with a highlight of the unit cell of the micro-structure.

Similarly, The determination of ψ for the nano-structure is defined by:

$$\psi_n = \left(L_{nano-edge} \times h_{nano} + A_p\right) \big/ A_p , \quad \text{(S8)}$$

where $L_{nano-edge}$ is the wetted perimeter of the nano-fishscale, h_{nano} is the height of the nano-fishscale (2.89 μm as shown in **Figure S9 (c)**) and A_p is the projected area. $L_{nano-edge}$ can be determined by a machine-assisted edge detection algorithm [7] using the area marked by the red dotted line in **Figure S9 (a)**. **Figure S9 (a)** typifies the nano-structured surface from the top view where the white areas represent the peaks of the grass and the dark regions represent the valleys. The area marked by the red dotted line, as presented in **Figure S9 (a)**, is selected for the edge detection algorithm as the scale bar would interfere the detection result. The detection result is demonstrated in **Figure S9 (b)**, with white lines demarcating the wetted perimeter of the nano-fishscale. By reviewing the scale bar in **Figure S9 (a)**, the length per pixel can be calculated to be 5.501 nm/pixel length. Therefore, $L_{nano-edge}$ was detected to be 50097 pixel-lengths, which corresponds to 275.583 μm. A_p of the marked area in **Figure S9 (a)** can be calculated to be 33.7 μm^2. Please refer to our work [8] uploaded in Zenodo for detailed materials, results and the edge detection code.

The solid fraction for the nano-micro surfaces can be determined by [9]:

$$\psi_{nm} = \psi_n \psi_\mu . \quad \text{(S9)}$$

ψ of different surfaces utilized in this paper are summarized in **Table S3**.

Table S3. Solid fraction of different surfaces.

Surfaces	Smooth	Micro	Nano	6-Nano-micro	100-Nano-micro
ψ	1.00	1.06	24.63	26.11	49.26

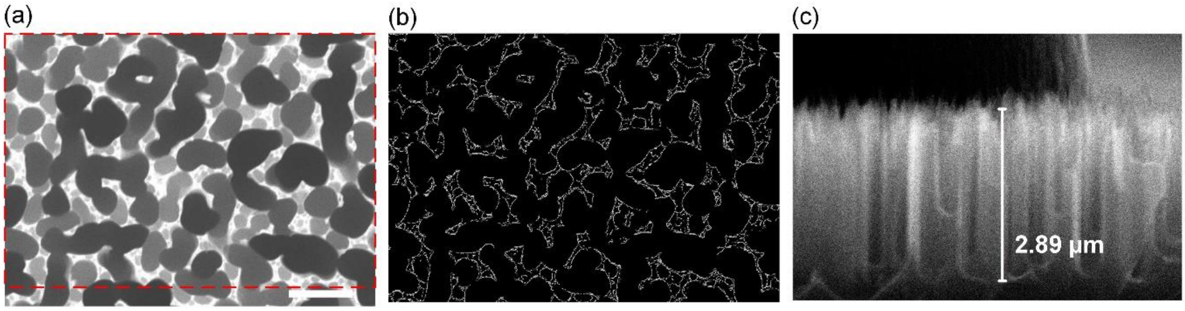

Figure S9. Characterization of the nano-structured surface. (a) top view of the nano-structured surface with a scale bar of 1 μm. The area marked by red dotted line is for edge detection algorithm. (b) Results of the edge detection upon the area marked by the red dotted line. (c) Height determination of the nano-fishscale. [8]

S5.5 Calculation of vapour pressure on micro-structured surfaces

The vapour pressure on micro-structured surfaces can be calculated by Eq. (3) where V_v and $V_{v,\mu}$ are obtained by Eqs. (S10) and (S11), respectively.

$$V_v = p_\mu^2 l_v \tag{S10}$$

$$V_\mu = p_\mu^2 l_v + h_\mu \left(p_\mu^2 - a^2 \right) \tag{S11}$$

Where h_v is the thickness of vapor layer, which is 2.5 μm [10].

S5.6 Calculation of capillary pressure of the HSS structure

The capillary pressure is defined by $P_{ca} = 2\sigma/l$ as displayed in the main body of the letter. For the HSS, l is the meniscus radius as calculated through **Figure S10** where l is obtained by $l = rh/(1-\sin\theta)$. According to **Figure S10**, l is calculated to be 3.0 mm. Therefore, P_{ca} for the 100-nano-micro is 43.3 Pa.

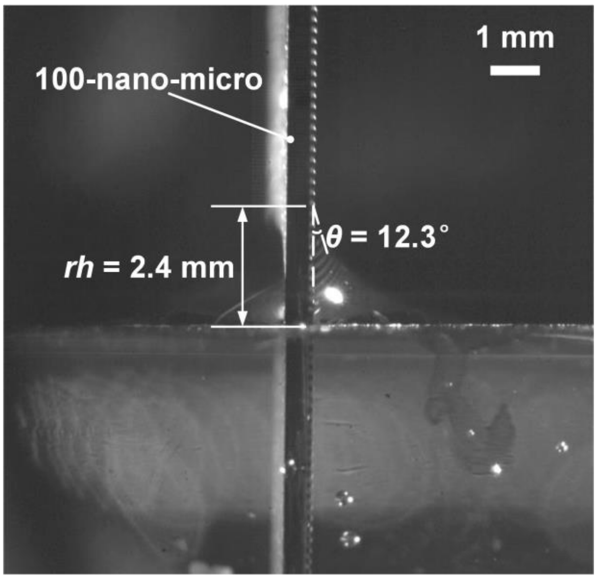

Figure S10. Meniscus radius measurement for the surface of 100-nano-micro.

S6 Reasons to establish DNN to model heat transfer patterns

Droplet boiling is influenced by a variety of physical mechanisms that depend on the operational conditions. For instance, when the wall temperature is below a specific threshold, the contact or nucleate boiling model prevails, whereas the Leidenfrost effect dominates once the temperature exceeds this threshold. Additional factors, including the presence of solid structures, droplet dynamics, and the state of the liquid film, can significantly influence the governing mechanism. Currently, there are no comprehensive models available that encompass the broad spectrum of conditions encountered in droplet boiling. As a result, the existing literature provides limited guidance for the design of droplet-based boiling heat transfer systems intended for diverse operating environments. DNN, a powerful information-processing framework, holds promise in bridging the gap between fundamental knowledge and practical applications of droplet boiling. The DNN-based approach, adept at handling the non-linear interactions governed by multi-physics and multiple parameters, has considerable potential to generate droplet boiling heat transfer data across a wide range of influencing factors. This could facilitate the development of precise and rapid predictive models and design guidelines for droplet-based cooling systems operating under varied conditions.

S7 Heat transfer database

Table S4. Droplet boiling heat transfer data.

We	Ψ	u'	T'	h (W/(m^2 K))
1.0	1.1	6.5	1.1	2451.2
1.0	1.1	6.5	0.8	1763.8
1.0	1.1	6.5	1.5	1743.3
1.0	1.1	6.5	0.6	925.9
1.0	1.1	6.5	1.8	345.8
1.0	26.1	6.0	1.8	485.3
1.0	26.1	6.0	1.5	2750.9
1.0	26.1	6.0	1.1	4447.2
1.0	26.1	6.0	0.8	3676.0
1.0	26.1	6.0	0.6	858.2
1.0	24.6	6.2	0.6	665.1
1.0	24.6	6.2	1.5	1941.4
1.0	24.6	6.2	0.8	3070.4
1.0	24.6	6.2	1.1	3138.9
1.0	24.6	6.2	1.8	144.3
1.0	1.0	9.9	1.1	1774.6
1.0	1.0	9.9	0.8	1584.1
1.0	1.0	9.9	1.5	1094.8

1.0	1.0	9.9	1.8	182.1
1.0	1.0	9.9	0.6	354.3
84.3	26.1	54.5	1.5	1923.8
84.3	26.1	54.5	0.8	2993.6
84.3	26.1	54.5	1.1	3867.9
84.3	26.1	54.5	1.8	921.6
84.3	26.1	54.5	0.6	1195.4
84.3	24.6	56.5	1.5	1822.6
84.3	24.6	56.5	0.8	2107.0
84.3	24.6	56.5	0.6	593.5
84.3	24.6	56.5	1.1	2607.6
190	1.0	134.3	1.1	1438.6
190	1.0	134.3	0.8	1371.9
190	1.0	134.3	1.5	1018.8
190	1.0	134.3	0.6	524.6
190	1.0	134.3	1.8	126.8
274	1.1	106.7	1.1	2139.1
274	1.1	106.7	0.6	1043.6
274	1.1	106.7	1.8	595.7
274	1.1	106.7	1.5	1446.3
274	1.1	106.7	0.8	2303.6
274	26.1	98.3	0.8	4249.3
274	26.1	98.3	1.8	1135.0
274	26.1	98.3	1.5	1923.8
274	26.1	98.3	1.1	4257.7
274	26.1	98.3	0.6	2555.1
274	24.6	101.9	0.8	3601.1
274	24.6	101.9	1.5	1703.1
274	24.6	101.9	1.8	654.7
274	24.6	101.9	1.1	2406.7
274	24.6	101.9	0.6	1214.2
274	1.0	162.2	1.1	1179.6
274	1.0	162.2	0.8	1828.5
274	1.0	162.2	0.6	710.7
274	1.0	162.2	1.5	983.3
274	1.0	162.2	1.8	88.9
274	49.3	36.6	1.1	4698.3
274	49.3	36.6	1.5	3591.1
274	49.3	36.6	1.8	2921.2
274	49.3	36.6	1.9	1698.1
274	49.3	36.6	2.1	330.5
1.0	49.3	2.2	1.1	3335.2
1.0	49.3	2.2	1.9	2705.1
1.0	49.3	2.2	2.1	2545.2
1.0	49.3	2.2	2.2	326.1
1.0	49.3	2.2	0.6	2362.0
1.0	49.3	2.2	0.8	3326.7
274	49.3	36.6	0.6	4650.9
274	49.3	36.6	0.8	3417.5

S8 Interim results of the active learning algorithm

Table S5. Count statistics of each saved training data point.

We	ψ	u'	T'	h (W/(m^2·K))	Counts
1.02	24.63	6.2227	1.8125	144.28	69
190	1.00	134.3063	0.5625	524.64	69
1.02	49.26	2.2366	2.225	326.06	69
190	1.00	134.3063	1.5000	1018.82	66
274	26.11	98.3129	1.5000	1923.80	65
1.02	26.11	6.0022	1.5000	2750.94	63
1.02	1.00	9.9044	0.5625	354.25	63
274	26.11	98.3129	1.8125	1135.04	63
274	24.63	101.9253	0.5625	1214.20	63
274	1.00	162.2296	1.0625	1179.65	63
274	49.26	36.6350	2.0625	330.52	63
84.3	26.11	54.5259	1.8125	921.57	62
190	1.00	134.3063	1.8125	126.79	62
274	24.63	101.9253	0.8125	3601.08	62
274	1.00	162.2296	1.8125	88.86	60
1.02	49.26	2.2366	1.9375	2705.09	60
274	49.26	36.6350	0.5625	4650.88	60
1.02	49.26	2.2366	2.0625	2545.19	60
1.02	1.06	6.5142	0.8125	1763.77	59
1.02	1.06	6.5142	1.5000	1743.33	59
1.02	1.06	6.5142	0.5625	925.90	59
1.02	24.63	6.2227	1.0625	3138.90	59
1.02	1.00	9.9044	1.0625	1774.59	58
84.3	26.11	54.5259	0.8125	2993.60	58
84.3	26.11	54.5259	0.5625	1195.41	58
190	1.00	134.3063	0.8125	1371.86	57
274	1.06	106.7003	0.5625	1043.63	57
274	26.11	98.3129	1.0625	4257.66	57
274	24.63	101.9253	1.0625	2406.74	57
274	1.00	162.2296	0.5625	710.74	57
1.02	1.06	6.5142	1.0625	2451.22	55
1.02	24.63	6.2227	0.5625	665.15	55
1.02	1.00	9.9044	0.8125	1584.11	55
1.02	1.00	9.9044	1.8125	182.06	55
84.3	26.11	54.5259	1.0625	3867.93	55
84.3	24.63	56.5294	1.5000	1822.63	55
84.3	24.63	56.5294	0.5625	593.51	55
190	1.00	134.3063	1.0625	1438.59	54
274	1.06	106.7003	1.8125	595.71	54
274	26.11	98.3129	0.8125	4249.28	54
274	1.00	162.2296	0.8125	1828.50	54
274	49.26	36.6350	1.9375	1698.15	54
1.02	49.26	2.2366	0.5625	2362.00	54
1.02	49.26	2.2366	0.8125	3326.73	54
274	49.26	36.6350	0.8125	3417.51	54
1.02	24.63	6.2227	0.8125	3070.45	51

1.02	1.00	9.9044	1.5000	1094.76	51
84.3	26.11	54.5259	1.5000	1923.80	51
274	1.06	106.7003	1.0625	2139.09	51
274	1.06	106.7003	1.5000	1446.30	51
274	26.11	98.3129	0.5625	2555.12	51
1.02	49.26	2.2366	1.0625	3335.22	51
274	1.06	106.7003	0.8125	2303.60	51
1.02	1.06	6.5142	1.8125	345.79	48
1.02	26.11	6.0022	1.0625	4447.15	48
1.02	26.11	6.0022	0.8125	3675.98	48
1.02	26.11	6.0022	0.5625	858.20	48
84.3	24.63	56.5294	0.8125	2107.00	48
274	24.63	101.9253	1.5000	1703.11	48
274	49.26	36.6350	1.5000	3591.07	48
1.02	24.63	6.2227	1.5000	1941.40	45
1.02	26.11	6.0022	1.8125	485.29	44
274	24.63	101.9253	1.8125	654.73	42
274	1.00	162.2296	1.5000	983.34	42
84.3	24.63	56.5294	1.0625	2607.65	39
274	49.26	36.6350	1.8125	2921.16	38
274	49.26	36.6350	1.0625	4698.25	36

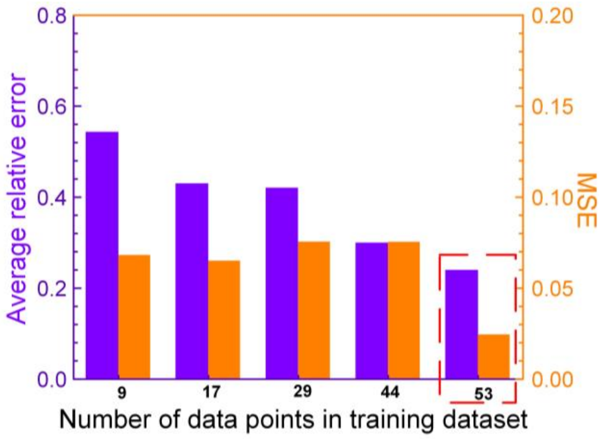

Figure S11. Effect of training data number on the prediction performances from the attention-integrated DNN. The average relative error $\bar{\delta}$ and mean square error (MSE) were estimated based on the prediction results in the testing datasets.

We selected five different quantities of training data points (9, 17, 29, 44, and 53) based on the frequency count in **Table S5**, which includes all experimental data from **Table S4**. During each model run, the data points not selected as the training data in **Table S5** were allocated to the testing dataset. For instance, if "44" was chosen as the number of training data points, the top 44 data points from **Table S5** would be included in the training dataset, while the remaining data points would be assigned to the testing dataset automatically. The prediction results from these varying quantities of training

data points are depicted in **Figure S11**. Generally, a larger training dataset yields better performance in terms of both error rate and mean squared error (MSE), adhering to the fundamental principle of the DNN model [11]. To minimize the scale of the training dataset, we balanced prediction performance against training dataset size. As illustrated in **Figure S11**, both the error rate and MSE increase sharply when the number of training data points falls below 53. Consequently, we settled on 53 as the optimal number of training data points, as highlighted by the red dotted rectangle in **Figure S11**, where the average relative error and MSE in the testing dataset are 21.2% and 0.01, respectively. The calculated average relative error and MSE were calculated by the average of one hundred runs of each models. Although the training data was the same for each run of a specific model, the prediction results for the one hundred runs varied due to the randomness of the weight of each neuron. The finalized optimized training dataset is provided in **Table S6**, and the remaining data, which automatically constitutes the testing dataset, is listed in **Table S7**.

Table S6. Data points in the optimized training dataset

We	ψ	u'	T'	h (W/(m^2·K))
1.02	24.63	6.2227	1.8125	144.28
190	1.00	134.3063	0.5625	524.64
1.02	49.26	2.2366	2.225	326.06
190	1.00	134.3063	1.5000	1018.82
274	26.11	98.3129	1.5000	1923.80
1.02	26.11	6.0022	1.5000	2750.94
1.02	1.00	9.9044	0.5625	354.25
274	26.11	98.3129	1.8125	1135.04
274	24.63	101.9253	0.5625	1214.20
274	1.00	162.2296	1.0625	1179.65
274	49.26	36.6350	2.0625	330.52
84.3	26.11	54.5259	1.8125	921.57
190	1.00	134.3063	1.8125	126.79
274	24.63	101.9253	0.8125	3601.08
274	1.00	162.2296	1.8125	88.86
1.02	49.26	2.2366	1.9375	2705.09
274	49.26	36.6350	0.5625	4650.88
1.02	49.26	2.2366	2.0625	2545.19
1.02	1.06	6.5142	0.8125	1763.77
1.02	1.06	6.5142	1.5000	1743.33
1.02	1.06	6.5142	0.5625	925.90
1.02	24.63	6.2227	1.0625	3138.90
1.02	1.00	9.9044	1.0625	1774.59
84.3	26.11	54.5259	0.8125	2993.60
84.3	26.11	54.5259	0.5625	1195.41
190	1.00	134.3063	0.8125	1371.86
274	1.06	106.7003	0.5625	1043.63

274	26.11	98.3129	1.0625	4257.66
274	24.63	101.9253	1.0625	2406.74
274	1.00	162.2296	0.5625	710.74
1.02	1.06	6.5142	1.0625	2451.22
1.02	24.63	6.2227	0.5625	665.15
1.02	1.00	9.9044	0.8125	1584.11
1.02	1.00	9.9044	1.8125	182.06
84.3	26.11	54.5259	1.0625	3867.93
84.3	24.63	56.5294	1.5000	1822.63
84.3	24.63	56.5294	0.5625	593.51
190	1.00	134.3063	1.0625	1438.59
274	1.06	106.7003	1.8125	595.71
274	26.11	98.3129	0.8125	4249.28
274	1.00	162.2296	0.8125	1828.50
274	49.26	36.6350	1.9375	1698.15
1.02	49.26	2.2366	0.5625	2362.00
1.02	49.26	2.2366	0.8125	3326.73
274	49.26	36.6350	0.8125	3417.51
1.02	24.63	6.2227	0.8125	3070.45
1.02	1.00	9.9044	1.5000	1094.76
84.3	26.11	54.5259	1.5000	1923.80
274	1.06	106.7003	1.0625	2139.09
274	1.06	106.7003	1.5000	1446.30
274	26.11	98.3129	0.5625	2555.12
1.02	49.26	2.2366	1.0625	3335.22
274	1.06	106.7003	0.8125	2303.60

Table S7. Data points in the testing dataset

We	ψ	u'	T'	*h* (W/(m^2·K))
1.02	1.06	6.5142	1.8125	345.79
1.02	26.11	6.0022	1.0625	4447.15
1.02	26.11	6.0022	0.8125	3675.98
1.02	26.11	6.0022	0.5625	858.20
84.3	24.63	56.5294	0.8125	2107.00
274	24.63	101.9253	1.5000	1703.11
274	49.26	36.6350	1.5000	3591.07
1.02	24.63	6.2227	1.5000	1941.40
1.02	26.11	6.0022	1.8125	485.29
274	24.63	101.9253	1.8125	654.73
274	1.00	162.2296	1.5000	983.34
84.3	24.63	56.5294	1.0625	2607.65
274	49.26	36.6350	1.8125	2921.16
274	49.26	36.6350	1.0625	4698.25

S9 Model validation using the 50-nano-micro

The surface of 50-nano-micro (ψ = 36.9) functions as validation of the established model in this letter. Its micro-pillar height is half of that of the 100-nano-micro but significantly higher than that of the 6-nano-micro. Therefore, the capacity of the vapor buffer channels (VBCs) for these three surfaces are obviously different with each other. **Figure S12** illustrates the wettability of the 50-nano-micro and comparisons among three utilized nano-micro HSSs. By comparison, the spreading ability of the 50-nano-micro is the best among the nano-micro HSSs. Especially when the contact time passes 50 ms, such superiority increases as the liquid upon the 100-nano-micro tends to move vertically instead of horizontally because of its high aspect ratio.

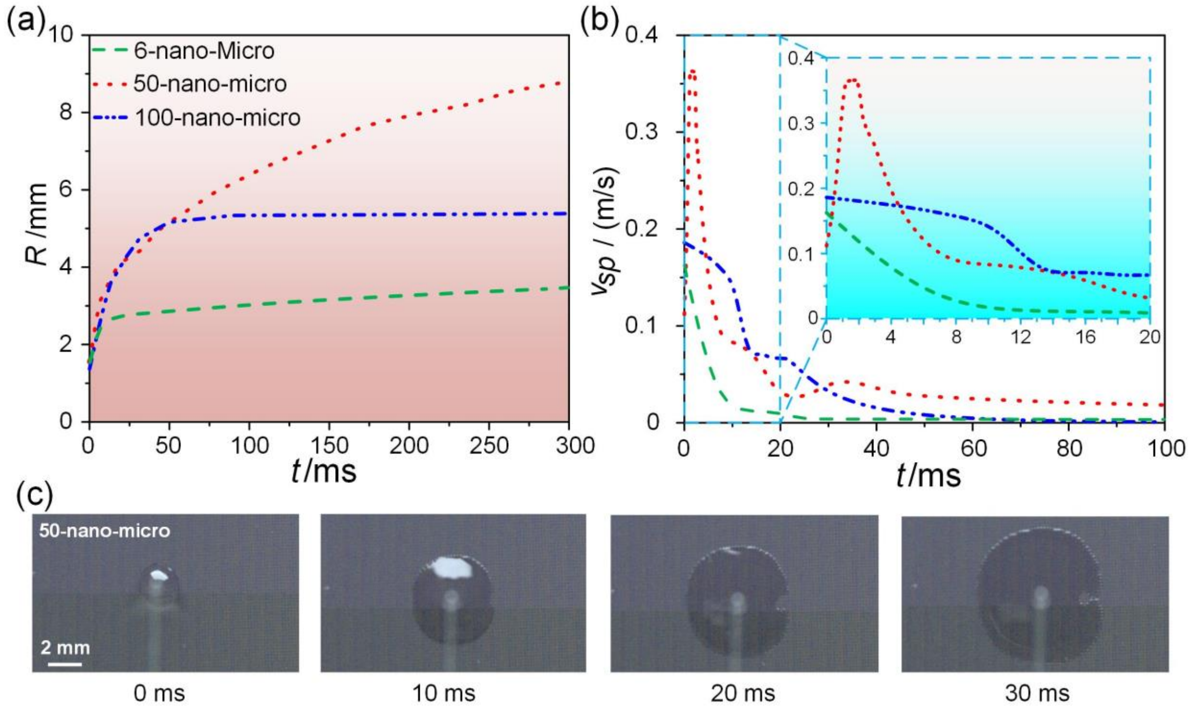

Figure S12. Wettability characterization of the 50-nano-micro surface. (a) The spreading radii (R) upon the utilized nano-micro HSSs. (b) Transient spreading velocities (v_{sp}) of these surfaces. (c) Wetting dynamic images of the 50-nano-micro surface. Data for the 6-nano-micro and 100-nano-micro in (a) and (b) functions as references.

The droplet boiling heat transfer data of the 50-nano-micro is listed in Table S8. As compared with those of the 6-nano-micro and 100-nano-micro, the heat transfer coefficient in high temperature region ($\dot{T} \geq 1.8$) of the 50-nano-micro is better than that of the 6-nano-micro but inferior to that of the 100-nano-micro which is coherent to rankings of the VBCs' capacity and ψ for these three surfaces. For We = 1.0, the LP occurs on the 255 ~ 265 ℃ 50-nano-micro, obtaining a 10 ~ 20 ℃ delay

compared to the 6-nano-micro. It shows the VBCs of 50-nano-micro do works in delaying the LP but such effect is not as obvious as the 100-nano-micro (the LP occurs on the 273 ℃ 10-nano-micro). The comparisons among these nano-micro HSSs validate the vapor buffer effect in delaying the LP.

Besides, the predicted h from the physics-informed attention-integrated DNN is compared with the experimental h as listed in Table S8. The average relative error is calculated to be 13.8%, validating the high accuracy of the trained DNN model. In addition, large relative errors always come from the high temperature region ($T' \geq 1.8$) where the absolute value of the experimental h is very small (near or at the LP). The prediction accuracy in the region of relative low-temperature region ($T' < 1.8$), where nucleate boiling occurs and common droplet cooling scheme operates, is rather high.

Table S8. Droplet boiling heat transfer validation data of the 50-nano-micro.

We	ψ	u'	T'	h (W/(m^2 K))	$\tilde{h}$ (W/(m^2 K))	Relative error
1.0	36.9	2.3	0.5625	2022.3	1983.9	-1.9%
1.0	36.9	2.3	0.8125	3248.4	3211.3	-1.1%
1.0	36.9	2.3	1.0625	3462.3	3455.3	-0.2%
1.0	36.9	2.3	1.5000	3206.2	3557.3	11.0%
1.0	36.9	2.3	1.8125	1768.7	2007.5	13.5%
1.0	36.9	2.3	1.9375	844.8	998.6	18.2%
1.0	36.9	2.3	2.0625	250.2	192.3	-23.1%
274	36.9	37.7	0.5625	4061.5	3758.8	-7.5%
274	36.9	37.7	0.8125	3766.1	3710.2	-1.5%
274	36.9	37.7	1.0625	3634.3	3537.3	-2.7%
274	36.9	37.7	1.5000	3401.7	3385.4	-0.5%
274	36.9	37.7	1.8125	896.8	1163.2	29.7%
274	36.9	37.7	1.9375	475.1	800.8	68.5%

S10 Limitations in heat transfer ability

Table S9. Comparison of LP temperatures among this work and previous publications.

References	[12]	[13]	[14]	This work	[15]	[16]
Solid Material	Homogeneous	Heterogeneous	Homogeneous	Homogeneous	Homogeneous	Heterogeneous
Coolant	Water	Water	Water	Water	Ice	Water
LP temperature (℃)	145	150	245	273	＞ 500	＞ 1000

In Table S9, it can be seen that the obtained LP temperature is not very high enough, which is, admittedly, a limitation in this paper (although the purpose of this paper is not to chase a higher LP temperature). As shown in Edalatpour et al.'s and Jiang et al.'s works [15][16], the current LP temperature can be elevated well above 500 ℃.

Comparison of heat transfer coefficients is listed in Table S10. According to Jiang's work, the current latest technology can obtain an extraordinary heat transfer coefficient (~ 2000 W/(m^2·K)) at a heterogeneous solid surface temperature above 1000 ℃. Our work demonstrates a good results (~ 4700 W/(m^2·K)) at the surface temperature below 200 ℃ and also remain a nucleate boiling with the heat transfer coefficient of around 1990 W/(m^2·K) at the surface temperature of 265 ℃, which is a decent performance for homogeneous solid materials.

Table S10. Comparison of heat transfer coefficients among this work and previous publications.

References	[12]	[14]		This work		[16]
Solid Material	Homogeneous	Homogeneous		Homogeneous		Heterogeneous
Surface type	Micro-structured	Nano-micro structured		Nano-micro structured		Nano-micro structured
Coolant type	Single water droplet	Single water droplet		Single water droplet		Multiple water droplet
Surface temperature (℃)	145	185	245	185	265	＞1000
Heat transfer coefficient (W/(m^2·K))	~ 410	~ 4430	~ 1110	~ 4700	~ 1990	~ 2000

S11 Further information about Videos S1−S3

Video S1: Boiling and fluid dynamics for droplet of *We* = 274 impinging on the 255 ℃ 100-nano-micro surface.

Video S2: Boiling and fluid dynamics for droplet of *We* = 1 impinging on the 265 ℃ 100-nano-micro surface.

Video S3: Boiling and fluid dynamics for droplet of *We* = 1 impinging on the 273 ℃ 100-nano-micro surface.

References

[1] M. Liu, S. Wang, Z. Wei, et al., Bioinspired design of a superoleophobic and low adhesive water/solid interface. Adv. Mater. 2009, 21(6), 665–669.

[2] A. R. Parker, C. R. Lawrence, Water capture by a desert beetle. Nature 2001, 414(6859), 33–34.

[3] R. Wen, X. Ma, Y.-C. Lee, and R. Yang, Liquid-vapor phase-change heat transfer on functionalized nanowired surfaces and beyond. Joule 2018, 2, 1–41.

[4] X. Yan, Y. Qin, F. Chen, G. Zhao, S. Sett, M.J. Hoque, K.F. Rabbi, et al., Laplace pressure driven single-droplet jumping on structured surfaces. ACS Nano 2020, 14, 12796–12809.

[5] J. Chen, R. Xu, Z. Zhang, X. Chen, X. Ouyang, G. Wang, P. Jiang, Phenomenon and mechanism of spray cooling on nanowire arrayed and hybrid micro/nanostructured surfaces. ASME J. Heat Transfer 2018, 140, 112401.

[6] J.-X. Wang, Y.-Z. Li, M.-L. Zhong, & H.-S. Zhang, Investigation on a gas-atomized spray cooling upon flat and micro-structured surfaces. Int. J. Therm. Sci. 2021, 161, 1106751.

[7] C. Ma, S. Kim, & N.X. Fang, Far-field acoustic subwavelength imaging and edge detection based on spatial filtering and wave vector conversion. Nat. Commun. 2019, 10, 204.

[8] J.-X. Wang, Z. Wu, & X. Chen, Quantification of the surface with nano-grass. Zenodo 2023. https://doi.org/10.5281/zenodo.7707565

[9] T. Maitra, M.K. Tiwari, C. Antonini, P. Schoch, S. Jung, P. Eberle, & D. Poulikakos, On the nanoengineering of superhydrophobic and impalement resistant surface textures below the freezing temperature. Nano Lett. 2014, 14, 172–182.

[10] T. Tran, H.J.J. Staat, A. Prosperetti, C. Sun, and D. Lohse, Phys. Rev. Lett. 2012, 108, 036101.

[11] S. Lee, S. Celik, B.A. Logsdon, et al., A machine learning approach to integrate big data for precision medicine in acute myeloid leukemia, Nat. Commun. 2018, 9, 1–13.

[12] J.-X. Wang, J. Qian, J.-X. Li, X. Wang, C. Lei, S. Li, et al., Enhanced interfacial boiling of impacting droplets upon vibratory surfaces. J. Colloid Interf. Sci. 2024, 658, 748–757.

[13] N. Farokhnia, S. M. Sajadi, P. Irajizad, H. Ghasemi, Decoupled hierarchical structures for suppression of Leidenfrost phenomenon. Langmuir 2017, 33(10), 2541–2550.

[14] J.-X. Wang, B. Cui, C. Salmean, X. Chen, X. Yan, Y. Mao, S. Yao, Machine-assisted quantification of droplet boiling upon multiple solid materials. Nano Energy 2024, 125, 109560.

[15] M. Edalatpour, C. L. Colón, J. B. Boreyko, Ice quenching for sustained nucleate boiling at large superheats. Chem 2023, 9, 1910–1928.

[16] M. Jiang, Y. Wang, F. Liu, H. Du, Y. Li, H. Zhang, et al., Inhibiting the Leidenfrost effect above 1,000 C for sustained thermal cooling. Nature 2022, 601(7894), 568–572.
